# Heterobimetallic Iridium-Niobia Catalyst for Efficient and Selective Methane Ammonia Reforming

**DOI:** 10.1021/jacs.6c04917

**Published:** 2026-06-15

**Authors:** Jiachun Wu, Zachary Dubrawski, Shiwen Wu, Samy Aïssiou, Lingzhe Fang, Laurent Veyre, Chloé Thieuleux, Tao Li, Clément Camp, Yizhi Xiang

**Affiliations:** † Department of Chemical and Biomedical Engineering, 14716 University of Missouri, Columbia, Missouri 65211, United States; ‡ Laboratory of Catalysis, Polymerization, Processes and Materials (CP2M UMR 5128), CNRS, Universite Claude Bernard Lyon 1, CPE-Lyon, Institut de Chimie de Lyon, 43 Bd du 11 Novembre 1918, F-69616 Villeurbanne, France; § Department of Chemistry and Biochemistry, 2848 Northern Illinois University, DeKalb, Illinois 60115, United States; ∥ X-ray Science Division, Argonne National Laboratory, Lemont, Illinois 60439, United States; ⊥ Department of Chemistry, Virginia Tech, Blacksburg, Virginia 24061, United States; # Materials Science and Engineering Institute, University of Missouri, Columbia, Missouri 65211, United States

## Abstract

A Surface OrganoMetallic Chemistry (SOMC) approach, leveraging a molecularly defined heterobimetallic niobium–iridium complex, was used to prepare a mesoporous SBA-15 silica-supported Ir-NbO_*x*_ catalyst. The resulting Ir-NbO_*x*_/SiO_2_ catalyst exhibited excellent catalytic performance in selective methane/ammonia reforming. Specifically, the Ir-NbO_*x*_/SiO_2_ catalyst showed significantly higher activity (turnover frequency 8.5 s^–1^), selectivity (75%), and stability than the Ir/SiO_2_ analog, whereas the NbO_*x*_/SiO_2_ counterpart was almost inactive. This contrasts with ethane/ammonia reforming via C–C cleavage, for which the bimetallic Ir-NbO_*x*_/SiO_2_ was less active than Ir/SiO_2_, demonstrating tuned selectivity toward C–H activation rather than C–C cleavage due to the Ir/NbO_*x*_ synergy. Importantly, an Ir-NbO_*x*_/SiO_2_ reference catalyst, prepared by conventional impregnation/calcination/reduction steps, was found to be inactive, highlighting the value of the SOMC catalyst preparation approach using well-defined heterobimetallic precursors. These results represent a significant advance over existing catalysts due to the atomic-scale synergy between Ir and NbO_*x*_ sites, enabling access to activity and selectivity regimes inaccessible to monometallic analogs.

## Graphical abstract

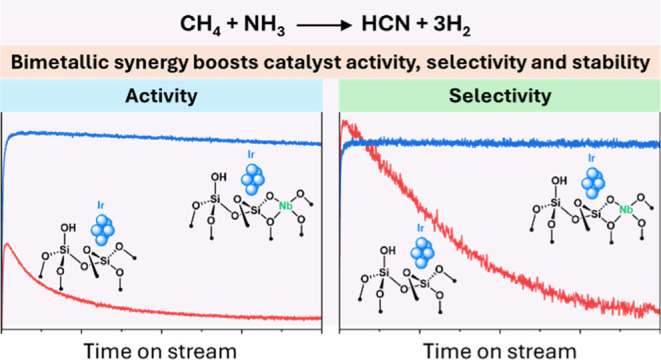

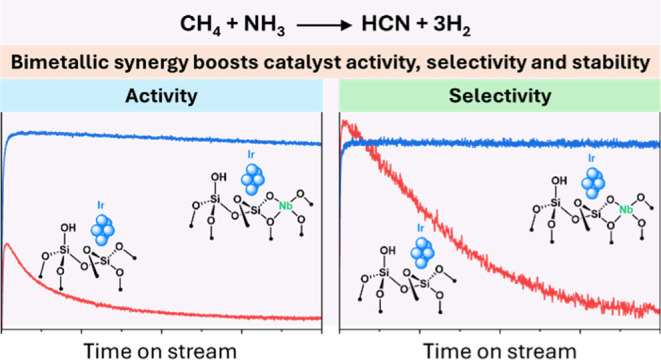

## Introduction

Atomic-scale synergy between heterobimetallic sites provides a unifying framework for transcending the intrinsic limitations of single-metal surfaces in heterogeneous catalysis. When two metal species are coupled at the atomic level, subtle electronic redistribution and local geometric reconfiguration collectively reshape the catalytic energy landscape, enabling reaction pathways and selectivity regimes inaccessible to monometallic analogs. Design strategies, such as impregnation,^1^ chemical reduction deposition,^2^ precipitation,^3^ electroless deposition,^4^ galvanic displacement,^5^ strong electrostatic adsorption,^6^ and surface organometallic chemistry (SOMC),^7–9^ have been developed over the past few decades, enabling control over the size and proximity of catalytically active, selective bimetallic sites.

Among the available approaches, the SOMC method is particularly powerful, involving the grafting of well-defined organometallic precursors onto hydroxylated supports, followed by controlled thermal treatments to generate isolated or paired bimetallic sites with uniform coordination environments. Owing to the predetermined metal–metal proximity inherent to the organometallic precatalysts, SOMC affords precise control over intermetallic distance and electronic interactions while minimizing particle-size heterogeneity. In recent years, Camp and co-workers have developed a series of dual-atom organometallic systems^10–17^ that were applied to the preparation of silica-supported bimetallic cluster or nanoparticle catalysts using the SOMC approach.^18–22^ These materials have demonstrated outstanding catalytic activity in C–H activation reactions,^21,23–25^ including methane and other light alkanes,^18,20,22^ exhibiting enhanced performances compared to monometallic analogs, along with higher selectivity for C–H activation over C–C bond cleavage. However, the application of these catalysts in industry-relevant reactions involving C–H activation and C–C cleavage has not been investigated.

An intriguing reaction with far-reaching implications for the chemical and energy industries is the ammonia (NH_3_) reforming of light alkanes for hydrogen cyanide (HCN) and dihydrogen production (eq 1),^26^ which follows a similar reaction stoichiometry as the conventional steam reforming.

1 $${\mathrm{CH}}_{4}+NH_{3}\to \mathrm{HCN}+3H_{2}$$

Conventional methane/ammonia reforming in industry, known as the Bläusaure aus Methan und Ammoniak (BMA) or Degussa process, is a well-established route for HCN production.^27,28^ A fundamental distinction between methane/ammonia reforming and other reforming reactions, such as steam or dry reforming, is that NH_3_ is intrinsically reactive and can be readily decomposed to N_2_ and H_2_ under reaction conditions, even in the absence of hydrocarbons. In particular, the average C–H bond energy in CH_4_ (414 kJ/mol) exceeds that of the N–H bond in NH_3_ (391 kJ/mol), rendering selective methane activation highly challenging. Additionally, most transition metal catalysts exhibit higher activity in NH_3_ decomposition than in CH_4_ activation. As a result, significant selectivity challenges often arise because NH_3_ has an inherent tendency to decompose into N_2_ and H_2_ when CH_4_ is not present in sufficient excess. However, operating under CH_4_ rich conditions promotes substantial coke formation. Among various transition metals, Pt has been identified as the most selective catalyst for methane/ammonia reforming by density functional theory (DFT) calculations from the Grabow and co-workers.^29^ However, the lower activity of Pt typically requires a higher reaction temperature, which explains why the BMA process was conventionally performed at temperatures above 1200 °C in alumina tube bundles coated with Pt-based catalyst.^30–32^ Other transition metals, such as Ir, Ni, and Co, although they were found to be close to the top of the volcano curve in activity, were predicted to have HCN selectivity below 50%.^29^ Consequently, low temperature (≤650 °C) ammonia reforming of light alkanes such as ethane or propane was not achieved until recent studies by the Xiang group using Re/HZSM-5 ^33^ or intermetallic Ni_3_Ga^34^ catalysts. However, for methane/ammonia reforming, the Re/HZSM catalyst was inactive, and the Ni_3_Ga catalyst exhibited lower NH_3_-based selectivity (<50%).^34^ Additionally, while Pt/HZSM-5 catalysts were found to be active at low temperatures for ammonia-assisted dehydrogenation to produce acetonitrile,^35,36^ it remains inactive in HCN production through ammonia reforming, as predicted by the DFT calculations.

The present study was motivated by the outstanding activity in selective methane C–H activation using heterobimetallic catalysts developed through the SOMC approach. We report the synthesis and characterization of a novel mesoporous SBA-15 silica-supported iridium-niobia (Ir-NbO_*x*_) catalyst and demonstrate its excellent catalytic performance for selective methane/ammonia reforming. Other transition metal heterobimetallic systems, such IrY, IrMo, and IrFe were also investigated during our initial catalyst screening, yet Ir-NbO_*x*_ demonstrated the best catalytic performance. The turnover frequency (TOF) and selectivity of HCN are up to 8.5 s^–1^ and 75%, respectively, significantly higher than the Ni_3_Ga catalyst, monometallic analogs, and the Ir-NbO_*x*_/SiO_2_reference catalyst (prepared by conventional impregnation/calcination/reduction steps), demonstrating the atomic scale synergy between the Ir and NbO_*x*_ sites and highlighting the value of the SOMC approach using well-defined heterobimetallic precursors.

## Results and Discussion

### Catalyst Preparation and Characterization

The synthesis and characterization of the starting heterobimetallic complex (Cp*IrH_2_)­(Cp*IrH_3_)_2_Nb­(NMe_2_), NbIr_mol_, from Cp*IrH_4_ and Nb­(NMe_2_)_5_ (Scheme 1) has been reported elsewhere.^37^ This complex was found to cleanly react with phenols through protonolysis of the dimethylamido ligand by releasing dimethylamine and forming a Nb–O bond, yielding the phenoxide-substituted analog (Cp*IrH_2_)­(Cp*IrH_3_)_2_Nb­(OAr).^17^ Accordingly, the heterobimetallic complex NbIr_mol_ can be grafted onto the surface of silica, since the silanol groups have similar acidity (p*K*_A_ ca. 7). Using such a SOMC approach, the surface-grafted complex, NbIr_*g*_/SiO_2_ (Scheme 1), was prepared through wet impregnation of SBA-15 (dehydroxylated at 700 °C under 10^–5^ mbar vacuum) with a pentane solution of complex NbIr_mol_ (concentration calculated to achieve 1 wt % Ir loading, corresponding to the consumption of ca. 20% of the available surface silanol groups). The obtained NbIr_*g*_/SiO_2_ is then treated under static dihydrogen (1 atm) at 500 °C to promote thermal cleavage of the hydrocarbon ligands, yielding the desired heterobimetallic Ir-NbO_*x*_/SiO_2_ catalyst. Elemental analysis finds the weight loadings of Ir and Nb to be 0.96% and 0.15%, respectively, corresponding to a 3.1:1.0 atomic ratio (consistent with Ir:Nb ratio in the NbIr_mol_ precursor), indicating that the two metals remain associated during the grafting process. The low carbon, nitrogen, and hydrogen contents following H_2_ thermal treatment confirm the successful removal of organic ligands, which was further supported by the absence of C–H stretching frequencies (expected to be around 3000 cm^–1^, see Figure S1) from the diffuse reflectance infrared Fourier transform (DRIFT) spectrum. For reference, the monometallic Ir/SiO_2_ and NbO_*x*_/SiO_2_ analogs were also prepared via a similar SOMC protocol using Cp*IrH_4_ and Nb­(NMe_2_)_5_, respectively (Scheme 1, bottom).

**Scheme 1:**
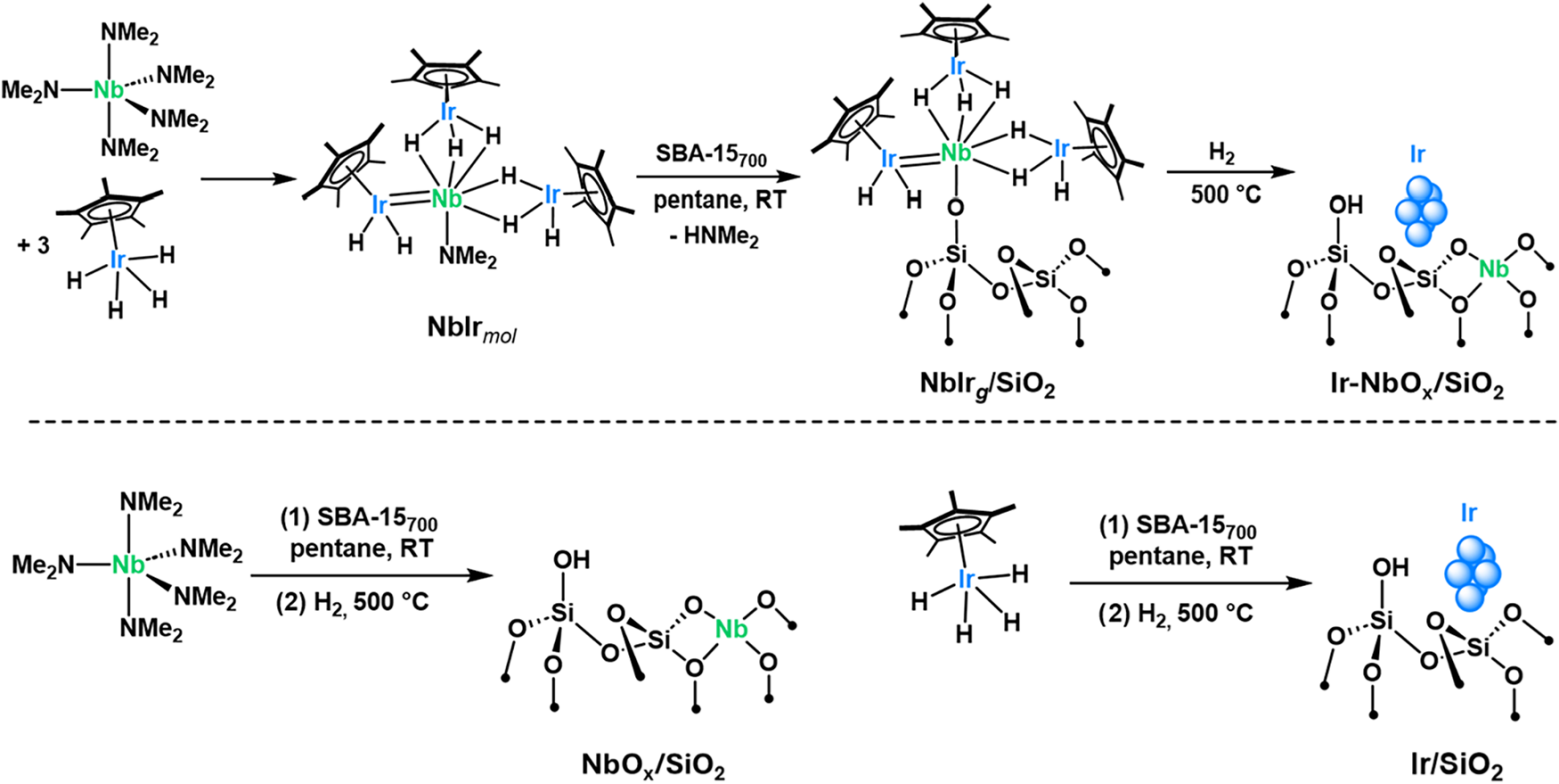
(Top) Preparation of the Niobium–Iridium Catalyst (Ir-NbO_*x*_/SiO_2_) via the SOMC Synthetic Approach; (Bottom) Preparation of the Monometallic NbO_*x*_/SiO_2_ and Ir/SiO_2_ Analogs Using a Similar SOMC Approach

The prepared catalysts were first characterized by high-angle annular dark-field scanning transmission electron microscopy (HAADF-STEM). As shown in Figure 1(a), subnanometer Ir particles/clusters were observed along the longitudinal pore channels of the SBA-15 support in both Ir-NbO_*x*_/SiO_2_ and Ir/SiO_2_ catalysts. This observation suggests that partial aggregation of the Ir component occurs during H_2_ reduction, in line with earlier studies of analogous systems.^18,22^ Energy-dispersive X-ray spectroscopy (EDS) analysis of catalyst regions corresponding to the HAADF-STEM images shown in Figures S2,S3 reveals overlapping spatial distributions of Nb and Ir in Ir-NbO_*x*_/SiO_2_, consistent with the intimate association of the two elements. While the STEM-EDS results do not provide sufficient spatial resolution to conclusively determine the presence or absence of phase-separated domains, isolated atoms, or monometallic species, they are consistent with a homogeneous distribution of Nb and Ir at the measurement’s spatial resolution. The mean Ir particle sizes, determined by H_2_ chemisorption (Figure S4), are 1.2 nm for Ir-NbO_*x*_/SiO_2_ and 1.4 nm for Ir/SiO_2_, in close agreement with the size histograms derived from STEM analysis. For the NbO_*x*_/SiO_2_ analog, HAADF-STEM images show no discernible particles or clusters, suggesting that Nb species are embedded within the silica matrix. In addition, X-ray diffraction (XRD) analysis of Ir-NbO_*x*_/SiO_2_, Ir/SiO_2_, and NbO_*x*_/SiO_2_ (Figure S5­(b)) reveals the absence of diffraction peaks corresponding to NbO_*x*_ or Ir phases, consistent with highly dispersed Nb and Ir species. Reference samples: Ir-NbO_*x*_/SiO_2_-ref (prepared from iridium­(III) chloride and niobium­(V) oxalate through impregnation/calcination/reduction) and Ir-NbO_*x*_/SiO_2_-TPO/TPR (the Ir-NbO_*x*_/SiO_2_ treated by temperature-programmed oxidation followed by reduction), were also characterized by XRD (Figure S5). Both samples exhibit distinct diffraction peaks corresponding to metallic Ir (111), Ir (200), and Ir (220), indicating the formation of relatively large Ir^0^ nanoparticles. In contrast, their precursors prior to reduction display diffraction peaks characteristic of rutile IrO_2_ (Figure S5­(a)),^38^ suggesting the formation of larger IrO_2_ nanoparticles during calcination or TPO treatment. This behavior is consistent with the tendency of IrO_2_ clusters to aggregate at temperatures ≥ 200 °C.

**Figure 1:**
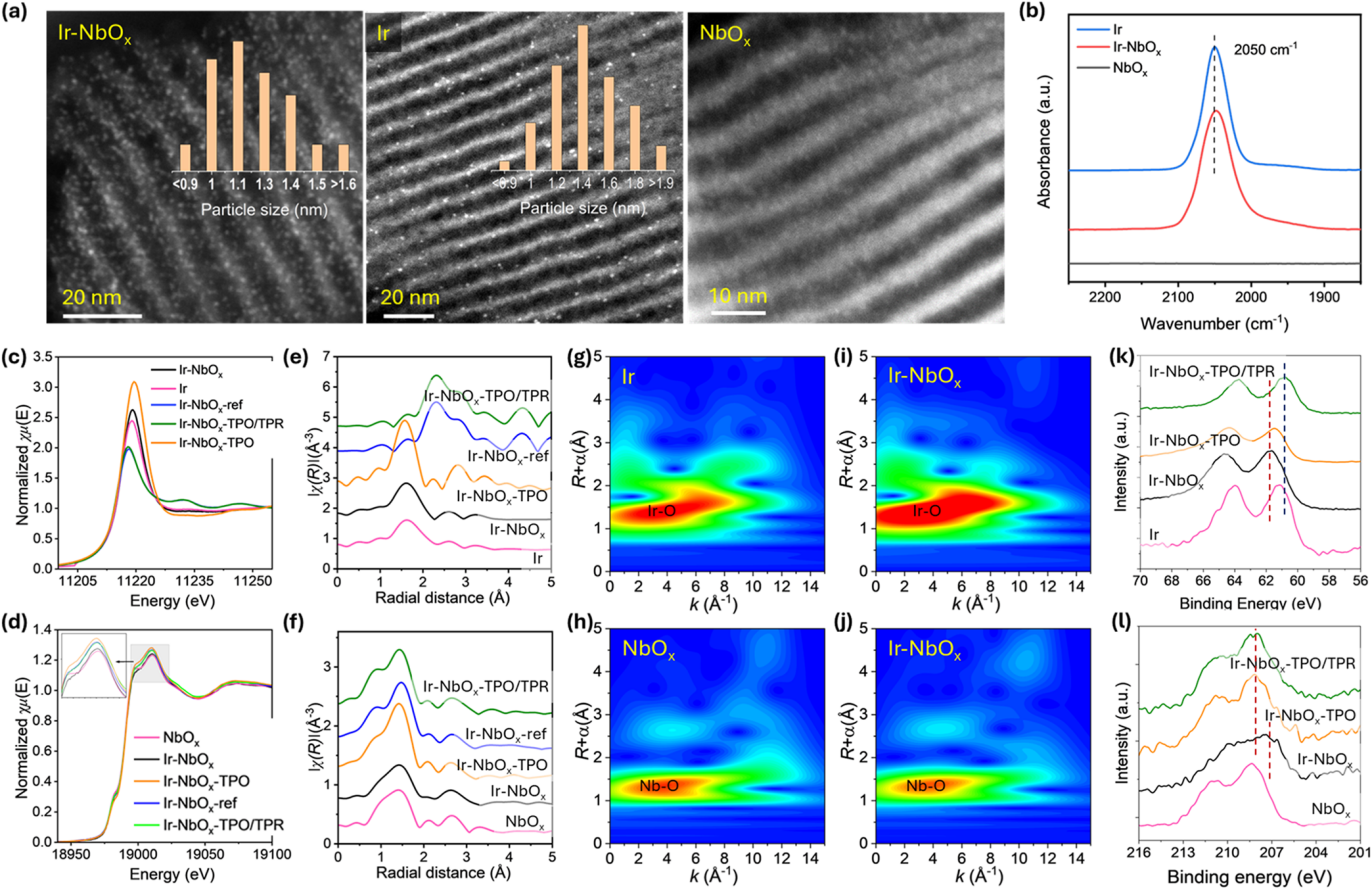
(a) Representative HAADF-STEM images of Ir-NbO_*x*_/SiO_2_, Ir/SiO_2_, and NbO_*x*_/SiO_2_. (b) CO adsorption DRIFT spectra. (c, d) normalized XANES spectra, (e, f) Fourier transform EXAFS spectra, (g–j) wavelet transform-EXAFS at Ir L_III_-edge and Nb K-edge, respectively. (k, l) Ir 4f and Nb 3d XPS spectra, respectively.

Carbon monoxide (CO) adsorption DRIFTS measurements were carried out to further characterize the prepared catalysts. As shown in Figure 1(b), both Ir-NbO_*x*_/SiO_2_ and Ir/SiO_2_ exhibit a well-defined C–O stretching band at ∼2050 cm^–1^, indicative of CO bound to low-coordination Ir sites (edges and corners) associated with small Ir clusters.^39,40^ These results further suggest that the nature of the Ir phase is similar in both catalysts and indicate the absence of alloying between Ir and Nb in Ir-NbO_*x*_/SiO_2_. Notably, no signal attributable to chemisorbed CO is detected for the NbO_*x*_/SiO_2_ analog, consistent with the inherently low CO affinity of early transition metals.^8,41–44^ Additionally, nitrogen physisorption results for the SiO_2_ support and the Ir-NbO_*x*_/SiO_2_ catalyst exhibit similar pore size distributions, with only a modest reduction in Brunauer–Emmett–Teller (BET) surface area and pore volume (Figure S6). This demonstrates that the support morphology is preserved during catalyst synthesis and that the SBA-15 mesostructure remains intact, in agreement with STEM analysis.

X-ray absorption spectroscopy (XAS) was used to further characterize the chemical states and coordination environments of the Ir and Nb sites in the prepared catalysts. Figure 1 (c) shows the X-ray absorption near-edge structure (XANES) spectra at the Ir L_III_-edge. The white-line features of both Ir-NbO_*x*_/SiO_2_ and Ir/SiO_2_ are intermediate between those of the Ir-NbO_*x*_/SiO_2_-TPO and Ir-NbO_*x*_/SiO_2_-TPO/TPR reference samples (assigned to IrO_2_ and Ir^0^, respectively, based on XRD), indicating that the Ir species are present in a partially oxidized ionic state.^45,46^ Ir-NbO_*x*_SiO_2_ exhibits a slightly higher white-line intensity than Ir/SiO_2_, indicating a strong interaction between Ir and NbO_*x*_ species.^47^ This observation is consistent with the slightly reduced Nb *K-edge* XANES spectrum (Figure 1(d)), in contrast to the Ir-NbO_*x*_/SiO_2_-TPO, suggesting the formation of partially reduced, nonstoichiometric niobia in the Ir-NbO_*x*_/SiO_2_ catalyst. From the k^2^-weighted Fourier-transform extended X-ray absorption fine structure (FT-EXAFS) spectra of the Ir L_III_-edge (Figure 1 (e)) and fitting (Figure S7 and Table S1), the first shell Ir–O coordination dominated Ir-NbO_*x*_/SiO_2_ and Ir/SiO_2_. For Ir/SiO_2_, the Ir–O coordination number (CN) at an R distance of 2 Å is around 4, dominating over the Ir–Ir coordination at 2.73 Å. Slightly higher Ir–O and Ir–Ir CN (both are around 6) were observed for the bimetallic Ir-NbO_*x*_/SiO_2_ catalyst. The high FT-EXAFS peak at 1.62 Å and the high Ir–O CN are consistent with the strong white lines in the XANES spectra. By contrast, the Ir-NbO_*x*_/SiO_2_-ref and Ir-NbO_*x*_/SiO_2_-TPO/TPR reference samples are dominated by Ir–Ir scattering features at *R* distance above 2Å, with CNs of 11.4 and 10.5, respectively. These results are in good agreement with the XRD detections of metallic Ir^0^ phases in both samples. Collectively, the XAS at the Ir L_III_-edge results suggested that the Ir species in the Ir/SiO_2_ and Ir-NbO_*x*_/SiO_2_ samples exist mainly as isolated atoms or small clusters, consistent with the absence of NbO_*x*_ and Ir diffraction in the XRD patterns. From the k^2^-weighted FT-EXAFS at Nb *K*-edge (Figure 1 (f)), the first shell Nb–O coordination dominated for Ir-NbO_*x*_/SiO_2_, NbO_*x*_/SiO_2_, and related reference samples, suggesting the formation of NbO_*x*_ species, which are embedded within the silica matrix, as suggested by the HAADF-STEM. Additionally, the wavelet transform (WT)-EXAFS spectra were plotted as contour lines (Figure 1(g–j)), again illustrating that the first-shell Ir–O and Nb–O coordination was dominant for the developed catalysts.

In addition to bulk XAS, X-ray photoelectron spectroscopy (XPS) was used to probe the surface electronic structure of the samples. As shown in Figure 1(k), the Ir 4f binding energies of Ir-NbO_*x*_/SiO_2_-TPO/TPR appear at 60.8 and 63.8 eV, consistent with metallic Ir^0^ (4f_7/2_ and 4f_5/2_). In contrast, Ir-NbO_*x*_/SiO_2_-TPO exhibits higher binding energy at 61.5 and 64.5 eV, characteristic of IrO_2_. The Ir/SiO_2_ sample displays intermediate binding energies (61.2 and 64 eV), indicating partially reduced Ir species. Notably, Ir-NbO_*x*_/SiO_2_ shows slightly higher Ir 4f binding energies than Ir-NbO_*x*_/SiO_2_-TPO, consistent with the presence of isolated atoms or subnanometer clusters, considering the particle size effect in XPS.^48^ The corresponding Nb 3d spectra (Figure 1(l)) show Nb 3d_5/2_ and 3d_3/2_ peaks at ∼208 and ∼211 eV for NbO_*x*_/SiO_2_, Ir-NbO_*x*_/SiO_2_-TPO, and Ir-NbO_*x*_/SiO_2_-TPO/TPR, consistent with Nb_2_O_5_. In contrast, Ir-NbO_*x*_/SiO_2_ exhibits a negative shift in Nb 3d binding energy, indicating partial reduction of niobia induced by Ir and a strong metal–support interaction. Taken together, XPS, XAS, XRD, and STEM characterizations indicate that the SOMC strategy affords exceptionally high metal dispersion and intimate electronic coupling between Ir and NbO_*x*_ species, which likely underpins the enhanced catalytic performance discussed below.

### Catalytic Results

The catalytic performances of Ir-NbO_*x*_/SiO_2_ and its monometallic analogs were first investigated for methane/ammonia reforming to demonstrate their activity in selective CH_4_ conversion (C–H activation). As shown in Figure 2, Ir-NbO_*x*_/SiO_2_ shows significantly higher activity, selectivity, and stability than the Ir/SiO_2_ monometallic counterpart, which rapidly deactivates after 1h TOS. NbO_*x*_/SiO_2_ by contrast shows no activity in methane/ammonia reforming and negligible activity in NH_3_ decomposition. In terms of activity, Ir-NbO_*x*_/SiO_2_ shows an initial CH_4_ conversion of 18.7% (Figure 2(a)), corresponding to a rate of HCN up to 15.1 mmol/g_cat_/min (Figure 2(d)) and TOF 8.5 s^–1^ based on H_2_ chemisorption; whereas the conversion and rate were only 8% and 6.5 mmol/g_cat_/min, respectively, for Ir/SiO_2_. In contrast, the Ir-NbO_*x*_/SiO_2_-ref and Ir-NbO_*x*_/SiO_2_-TPO/TPR reference catalysts exhibit poor performance in methane/ammonia reforming, with CH_4_ conversion below 2% and HCN rate below 3 mmol/g_cat_/min. This low activity is attributed to the formation of large metallic Ir^0^ particles during the calcination/reduction, as evidenced by the characterization results discussed above. These findings underscore the advantage of the SOMC strategy, which enables the generation of highly dispersed, intimately coupled Ir-NbO_*x*_ species via direct thermal decomposition of well-defined heterobimetallic precursors. Notably, the HCN formation rate over Ir-NbO_*x*_/SiO_2_ catalyst is significantly higher than that over the Ni_3_Ga catalyst, which exhibits a rate of only 1.05 mmol/g_cat_/min.^34^ Additionally, the Pt/Al_2_O_3_ (1 wt % Pt loading) catalyst (employed for the high-temperature BMA process) was found to be inactive (see Figure S8), with CH_4_ conversion below 1%, under the investigated low-temperature reaction conditions.

**Figure 2:**
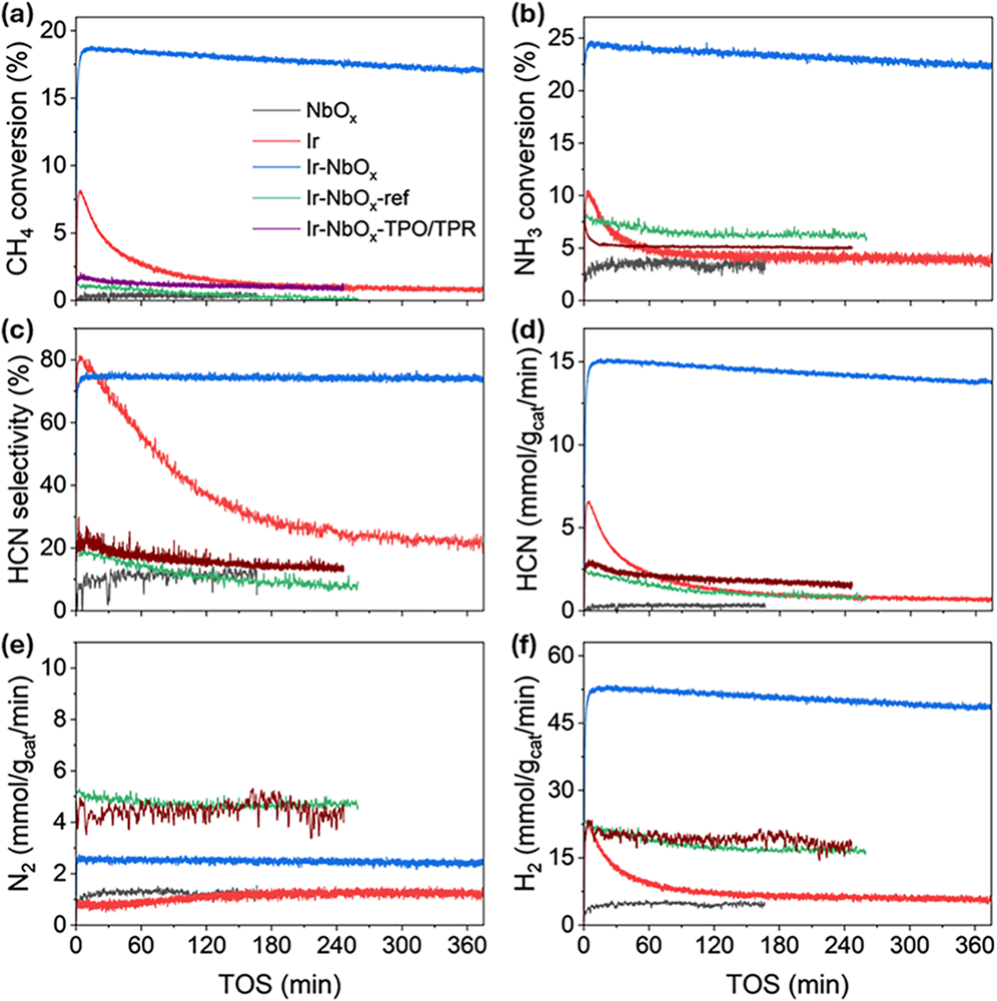
Catalytic performance of Ir-NbO_*x*_/SiO_2_ catalyst and its monometallic analogs (Ir/SiO_2_ and NbO_*x*_/SiO_2_) prepared by SOMC in methane/ammonia reforming. The performance of the NbIr/SiO_2_-ref and Ir-NbO_*x*_/SiO_2_-TPO/TPR catalysts is also plotted for comparison. Reaction conditions: 650 °C, m_catalyst_ = 10 mg, space velocity 480,000 mL/g_cat_/h, partial pressure of CH_4_/NH_3_/Ar: 0.25/0.25/0.5 atm.

For all investigated catalysts, NH_3_ conversion (Figure 2(b)) exceeds CH_4_ conversion (Figure 2(a)), indicating that a portion of NH_3_ is decomposed into N_2_ and H_2_, or some HCN is hydrogenated to form CH_4_ and N_2_ via reaction, 2HCN + 3H_2_→2CH_4_ + N_2_, (see Figure 2(e) N_2_ formation rates). These parasitic side reactions are primary concerns in the commercial BMA process. HCN selectivity was calculated as the molar ratio of NH_3_ converted to HCN relative to the total converted NH_3_. As shown in Figure 2(c), HCN selectivity remains around 75% over the Ir-NbO_*x*_/SiO_2_ catalyst with time-on-stream (TOS) up to 360 min, outperforming the Ni_3_Ga catalyst (47.7%) previously reported.^34^ Considering the “lower” reaction temperature (650 °C in contrast to ≥ 1200 °C for the industry BMA process) employed in this study, 75% is a high HCN selectivity, although direct comparison with other literature is limited due to the absence of studies at “lower” temperatures. Typically, high selectivity of methane/ammonia reforming was realized at low NH_3_ partial pressure and high temperature.^49^

With respect to stability, Ir-NbO_*x*_/SiO_2_ exhibits only slight deactivation over 360 min on stream, whereas Ir/SiO_2_ undergoes pronounced deactivation under identical conditions. As shown in Figure 2 (d,e), both HCN and N_2_ formation rates over Ir-NbO_*x*_/SiO_2_ decrease modestly and with comparable deactivation constants, resulting in an essentially constant HCN selectivity (Figure 2(c)). These trends suggest that methane/ammonia reforming and NH_3_ decomposition proceed on the same catalytically active sites. The extended stability and regenerability of the Ir-NbO_*x*_/SiO_2_ catalyst were evaluated with TOS up to 150 h (Figure 3(a)). Although a modest decrease in initial activity was observed, catalyst stability improved after regeneration in 10% H_2_/Ar at the reaction temperature for 1 h. Notably, nearly identical catalytic performance was obtained during the third (TOS = 108–128 h) and fourth (TOS = 130–150 h) cycles, demonstrating excellent regenerability and underscoring the potential industrial applicability of the Ir-NbO_*x*_/SiO_2_ catalyst. However, the HCN rate decreased significantly, while the N_2_ rate remained almost constant with TOS over the Ir/SiO_2_ catalyst. Therefore, while the initial HCN selectivity reached 80%, it decreased to less than 30% after 160 min of TOS. The fast deactivation of the monometallic Ir/SiO_2_ catalyst is likely due to the reduction and sintering of the Ir sites (*vide infra*). Finally, the rate of H_2_ formation is shown in Figure 2(f) and corresponds well with the rates of HCN and N_2_.

**Figure 3:**
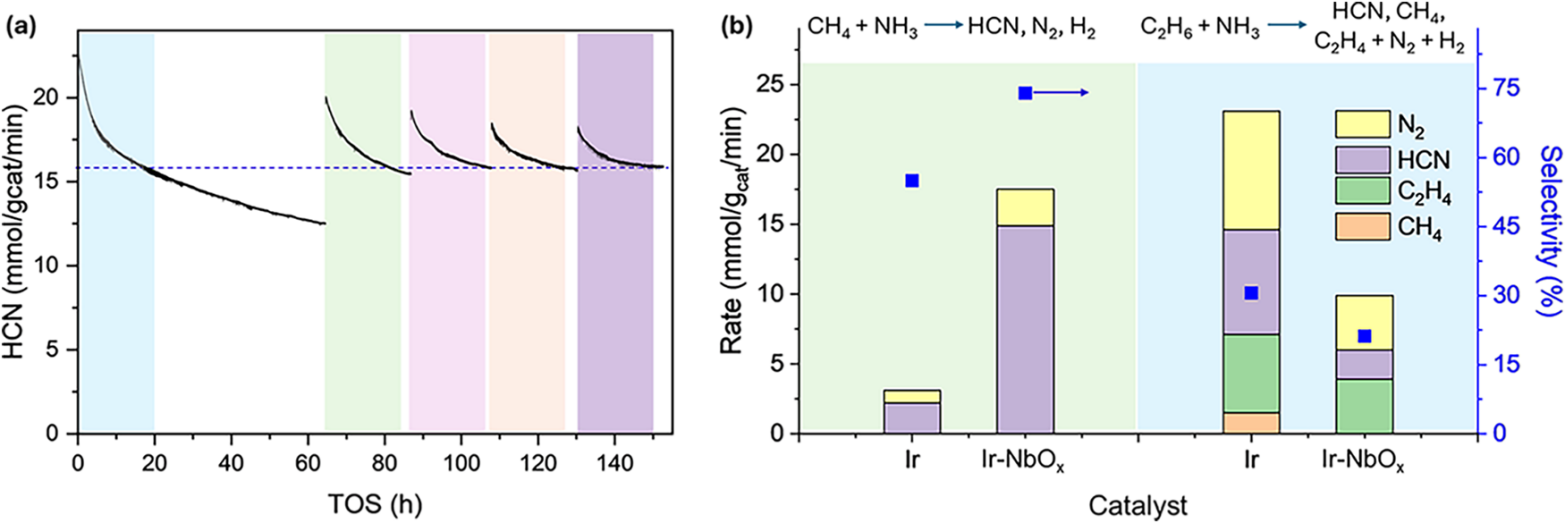
(a) Extended stability and regenerability of the Ir-NbO_*x*_/SiO_2_ catalyst in methane/ammonia reforming. (b) Rate of different products and ammonia-based selectivity obtained at TOS of 60 min during both methane and ethane ammonia reforming over the Ir-NbO_*x*_/SiO_2_ and Ir/SiO_2_, respectively. Regeneration was performed by switching the feed from reactants to 10% H_2_/Ar at 650 °C and holding for 1 h, and see Figures 2 and S9 for detailed reaction conditions.

The Ir-NbO_*x*_/SiO_2_ and Ir/SiO_2_ catalysts were further investigated for ethane/ammonia reforming to assess their activity in C_2_H_6_ conversion, which requires both C–H activation and C–C bond cleavage. Under these conditions, CH_4_ and C_2_H_4_ were detected as byproducts, arising from ethane hydrogenolysis (or HCN hydrogenation) and ethane dehydrogenation, respectively. Interestingly, as shown in Figures S9 (a–c) and 3(b), the bimetallic Ir-NbO_*x*_/SiO_2_ catalyst exhibits a substantially lower HCN formation rate (<3 mmol/g_cat_/min) during ethane/ammonia reforming than that observed in methane/ammonia reforming. Moreover, no byproduct CH_4_ is detected, indicating that the catalyst is essentially inactive in C–C cleavage. Additionally, C_2_H_4_ and N_2_ formation rates are higher than the HCN formation rate, corresponding to lower HCN selectivity (∼20% for both ethane- and ammonia-based selectivity). The lower selectivity stemmed mainly from a lower rate of HCN formation (C–C cleavage) rather than a higher rate of byproduct formation. Notably, the rate of N_2_ formation during methane/ammonia reforming is only slightly lower than that from ethane/ammonia reforming. In contrast, the monometallic Ir/SiO_2_ catalyst exhibits a higher HCN formation rate during ethane/ammonia reforming than the bimetallic Ir-NbO_*x*_/SiO_2_ catalyst, and even exceeds its own activity observed during methane/ammonia reforming. Specifically, as shown in Figure S10, the early stage (TOS < 1 min) rates of CH_4_ and HCN formation were up to 58 and 21 mmol/g_cat_/min, respectively, indicating that the fresh Ir/SiO_2_ was highly active for C–C cleavage. However, this activity decays extremely rapidly with TOS. After 25 min (Figure S9 (d–f)), a comparatively stable catalytic regime is established, indicating the presence of two distinct deactivation mechanisms operating on different time scales. The rates at a TOS of 60 min indicated that the Ir/SiO_2_ catalyst is neither active nor selective in either methane or ethane ammonia reforming (Figure 3(b)). In contrast, the bimetallic Ir-NbO_*x*_/SiO_2_ catalyst was highly active, selective, and relatively stable in methane/ammonia reforming, highlighting its superior performance for C–H bond activation.

### Kinetics and Discussion

The influence of reaction conditions on the catalytic performance of the Ir-NbO_*x*_/SiO_2_ in methane/ammonia reforming was systematically investigated. As shown in Figure 4(a,b), at a constant NH_3_ partial pressure of 0.5 atm, CH_4_ conversion first increased, then decreased; NH_3_ conversion initially decreased, then remained almost constant, and HCN selectivity increased with increasing CH_4_ partial pressure. On the other hand, at a constant CH_4_ partial pressure of 0.25 atm, NH_3_ conversion decreased, CH_4_ conversion and HCN selectivity increased with increasing NH_3_ partial pressure (Figure 4(d,e)). Additionally, the influence of CH_4_ and NH_3_ partial pressures on the rates of HCN and N_2_ formation (Figure 4(c,f)) suggested a Langmuir–Hinshelwood mechanism for both methane/ammonia reforming and ammonia decomposition. Based on the hypothesized mechanism, the derived apparent rate equations are shown in Figure 4(j). The calculated rates of HCN and N_2_ formation, based on the parameters from the nonlinear regression of the rate equation, matched the experimentally obtained rates (see also Table S2).

**Figure 4:**
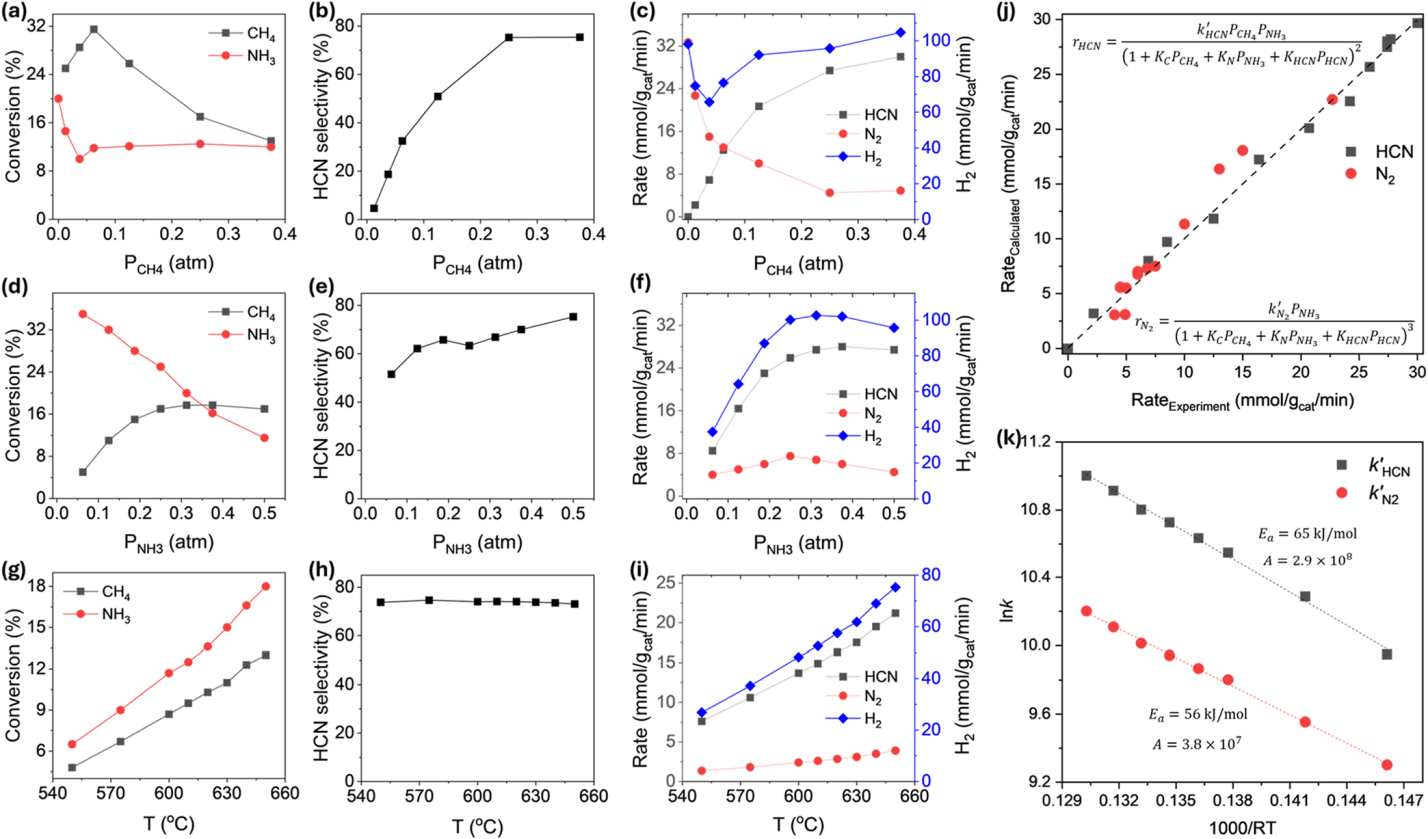
Influence of reaction conditions and kinetics on methane/ammonia reforming over the Ir-NbO_*x*_/SiO_2_ catalyst. (a–c) Influence of methane partial pressure at 650 °C and ammonia partial pressure of 0.5 atm. (d–f) Influence of ammonia partial pressure at 650 °C and methane partial pressure of 0.25 atm. (g–i) Influence of temperature at partial pressure of CH_4_/NH_3_/Ar: 0.25/0.25/0.5 atm. All data were collected at a time-on-stream of approximately 5 min, corresponding to steady-state reaction conditions without detectable catalyst deactivation. Other conditions: m_catalyst_ = 10 mg, space velocity 960,000 mL/g_cat_/h. (j) Calculated rates of HCN and N_2_ formation based on the developed rate equation versus experimental rates from panels (c, f). (k) Arrhenius plots of apparent rate constants of HCN and N_2_ formation.

The temperature-dependent catalytic performance of Ir-NbO_*x*_/SiO_2_ is shown in Figure 4(g–i). The conversion of CH_4_ and NH_3_, along with the formation rate of HCN and N_2_, increased exponentially with increasing temperature from 550 to 650 °C; whereas the selectivity remained essentially unchanged. Apparent rate constants, *k*′_HCN_ and *k*′_N2_, were extracted from the rate expressions (see Table S3), and the corresponding Arrhenius plots are shown in Figure 4(k). The apparent activation energies are 65 kJ/mol for HCN formation and 56 kJ/mol for N_2_ formation. The lower activation energy for N_2_ formation is offset by a smaller pre-exponential factor, indicative of the kinetic compensation effect.^50^ As a result, the product selectivity remained essentially invariant with temperature, despite the difference in activation energies.

To gain mechanistic insight, operando DRIFTS measurements were performed to identify surface species formed under reaction conditions. As shown in Figures 5(a) and S11, both NH_3_ and CH_4_ are adsorbed on the catalyst surface. The band at 3432 cm^–1^ is assigned to chemisorbed NH_3_ on the SiO_2_ support, supported by the concomitant negative silanol (Si–OH) features at 3737 cm^–1^. In addition, bands at 1552 cm^–1^ corresponding to NH_2_ species indicate activation of NH_3_ at catalytically active sites, while weaker features at 3040 and 3140 cm^–1^ are attributed to activated NH_*x*_ and CH_*x*_ species (Figure S11). Notably, intense bands at 2029 and 2050 cm^–1^ are observed and assigned to surface cyanide species, including chemisorbed HCN and related intermediates. These species are strongly bound to the surface and require up to 1 h to desorb under inert conditions. Compared to Ir/SiO_2_, the Ir-NbO_*x*_/SiO_2_ catalyst exhibits higher cyanide band intensity and a shift to higher wavenumber, indicative of electronic interactions between Ir and NbO_*x*_ sites.

**Figure 5:**
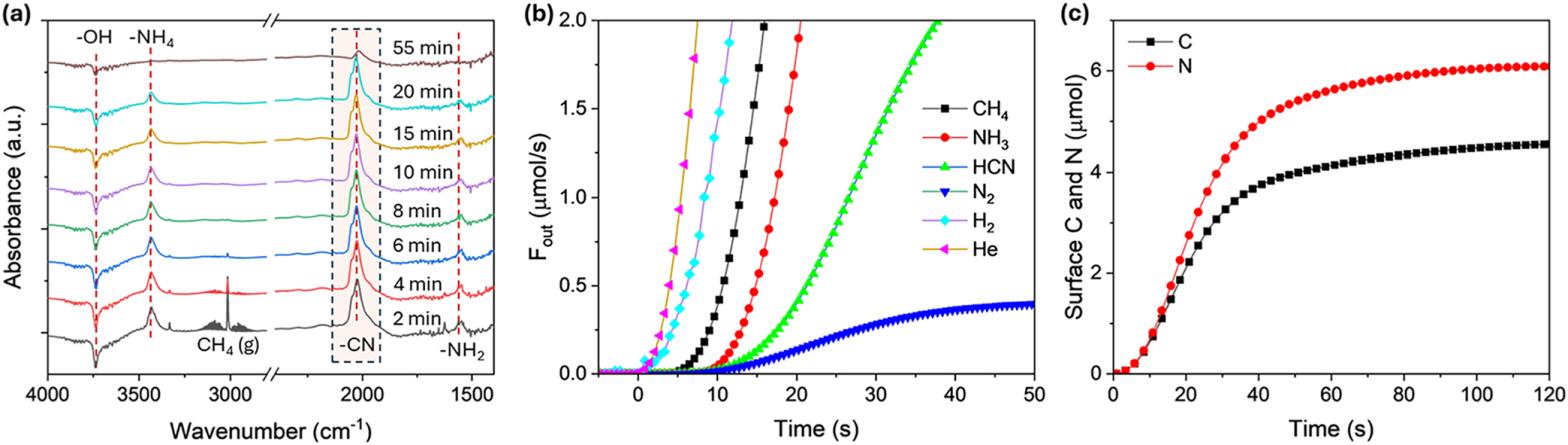
Operando DRIFT spectroscopy (a) and transient kinetic analysis ((b, c)) of methane/ammonia reforming on Ir-NbO_*x*_/SiO_2_ catalyst. (a) DRIFT spectra at 600 °C after switching from reactants (steady-state reaction conditions) to inert at different times; (b) reactor outlet flow during the early stage induction period; (c) catalyst surface C and N species accumulation during the early stage induction period. The transient experiment was performed at 650 °C on 0.02 g of Ir-NbO_*x*_/SiO_2_ catalyst with a CH_4_/NH_3_/He flow of 20/20/40 mL/min.

The spectroscopic observations were corroborated by quantitative transient kinetic analysis. The reactor outlet profiles during the early induction period are shown in Figure 5(b). Both CH_4_ and NH_3_ display clear delays relative to He, indicating adsorption on the catalyst surface. In contrast, N_2_ and HCN exhibit longer delay times, suggesting that surface reconstruction precedes their formation. Meanwhile, H_2_ appears concurrently with He and approximately 10 s prior to HCN formation, indicating that C–H and N–H bond dissociation occurs during the early stages of surface evolution. Quantitative mole balance analysis (Figure 5(c)) reveals surface coverages of around 4.5 μmol of carbon and 6 μmol of nitrogen, in good agreement with the operando DRIFTS observation of abundant −CN and NH_*x*_ species. Taken together, the operando DRIFTS and transient kinetic analyses support a Langmuir–Hinshelwood mechanism involving coadsorption, surface reconstruction, and coupling of C- and N-containing intermediates.

The used Ir-NbO_*x*_/SiO_2_ and Ir/SiO_2_ catalysts (after ∼6 h of steady-state methane/ammonia reforming) were further characterized by HAADF-STEM and XAS. As shown in Figure 6(a), negligible sintering was observed for the spent Ir-NbO_*x*_/SiO_2_ catalyst, whereas the Ir particle size in the spent Ir/SiO_2_ slightly increased (see also Figure S12). Sintering of Ir nanoparticles, therefore, could contribute to the deactivation of Ir/SiO_2_. XRD analysis of the spent Ir-NbO_*x*_/SiO_2_ and Ir/SiO_2_ catalysts (Figure S13) shows no diffraction features attributable to NbO_*x*_ or Ir phases, consistent with the fresh samples. This observation indicates the absence of significant sintering under reaction conditions, in agreement with the corresponding STEM results. EDS analysis shows Ir loadings comparable to those of the fresh samples, indicating that neither volatilization nor leaching of Ir takes place under the reaction conditions. Notably, no carbon nanofibers were observed in either spent catalyst by STEM, and temperature-programmed oxidation revealed CO_2_ evolution below the mass spectrometric detection limit. Furthermore, the C 1s XPS spectra of the spent Ir/SiO_2_ and Ir-NbO_*x*_/SiO_2_ catalysts are essentially identical to those of the fresh catalysts (Figure S14). These results indicate that coke formation is negligible during methane/ammonia reforming, consistent with our previous study on the Ni_3_Ga catalyst.^34^ The N 1s XPS spectrum of the spent Ir-NbO_*x*_/SiO_2_ exhibits a pronounced peak at ∼398 eV (Figure S15), indicating partial nitridation of the NbO_*x*_ surface during reaction, which is consistent with the subtle increase in the Nb 3d signal (Figure S16) at binding energies of 205–207 eV, characteristic of NbO_*x*_N_*y*_ species.^51^

**Figure 6:**
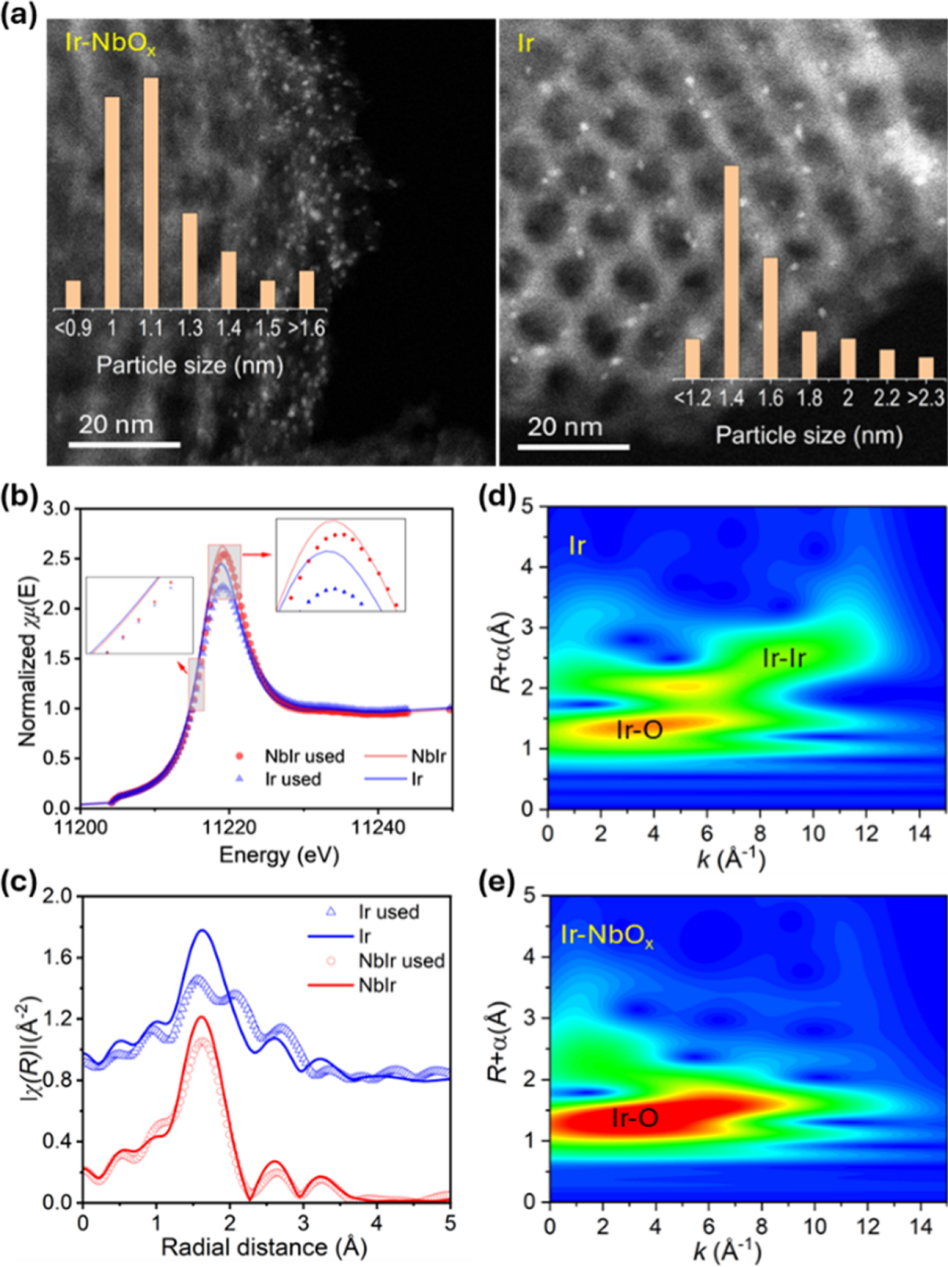
(a) Representative HAADF-STEM images, (b–e) Ir L_III_-edge XAS analysis: (b) normalized XANES spectra, (c) Fourier transform EXAFS spectra, (d–e) wavelet transform-EXAFS of the used Ir-NbO_*x*_/SiO_2_ and Ir/SiO_2_ catalysts.

The XANES spectra of the spent Ir-NbO_*x*_/SiO_2_ and Ir/SiO_2_ catalysts showed slightly decreased white lines in contrast to the catalyst before reaction (Figure 6 (b)), indicating that the catalysts were slightly reduced during the reaction. Importantly, from the k^2^-weighted FT-EXAFS spectra (Figure 6 (c)) and fitting (Figure S17 and Table S1) of the spent catalysts, the CN of Ir–O decreased, and Ir–Ir increased for the spent Ir/SiO_2_ in contrast to the fresh analog, whereas the spent bimetallic Ir-NbO_*x*_/SiO_2_ showed a similar FT-EXAFS spectrum and Ir–O/Ir-Ir CN to the fresh analog. Additionally, the WT-EXAFS spectra at Ir L_III_-edge of the spent catalysts, shown in Figure 6 (d,e), again illustrated that the Ir–Ir coordination increased for the Ir/SiO_2_ but remained similar for the Ir-NbO_*x*_/SiO_2_ after reaction. Nonetheless, the Ir 4f X-ray photoelectron spectroscopy (XPS) spectrum of the spent Ir-NbO_*x*_/SiO_2_ catalyst (Figure S18) exhibits a slight negative shift in binding energy relative to the fresh sample, indicating a modification of the Ir-NbO_*x*_ surface interaction. This shift is likely associated with partial nitridation of the NbO_*x*_ surface during the reaction. Finally, the XANES and FT-EXAFS spectra collected at the Nb K-edge for the fresh and spent Ir-NbO_*x*_/SiO_2_ catalyst are shown in Figure S19. The Nb coordination environment after reaction is essentially identical to that of the fresh catalyst, indicating that reaction-induced nitridation is confined to the near-surface region and remains below the detection limit of this bulk-sensitive technique.

Taken together, characterization results of the spent catalysts, combined with the stability and regenerability catalytic data, indicate that reaction-induced sintering leading to irreversible deactivation is negligible. Notably, sintering is particularly suppressed in Ir-NbO_*x*_/SiO_2_ due to the presence of Nb, consistent with a strong Ir-NbO_*x*_ interaction. Although partial surface nitridation of NbO_*x*_ is observed under reaction conditions, these subtle surface modifications do not result in irreversible catalyst deactivation. Importantly, the deactivated catalyst can be readily regenerated by treatment under 10% H_2_/Ar to reverse the reaction-induced surface modifications.

## Conclusions

Heterobimetallic iridium-niobia (Ir-NbO_*x*_) catalyst and its monometallic counterparts were prepared by grafting organometallic (Cp*IrH_2_)­(Cp*IrH_3_)_2_Nb­(NMe_2_), Cp*IrH_4_, and Nb­(NMe_2_)_5_ precursors onto dehydroxylated mesoporous SBA-15 silica using Surface Organometallic Chemistry (SOMC) approach, followed by dihydrogen thermal reduction to remove the organic ligands. The resulting Ir-NbO_*x*_/SiO_2_, Ir/SiO_2_, and NbO_*x*_/SiO_2_ catalysts showed well-dispersed subnanometer Ir clusters with Nb sites incorporated at the surface of the silica support. Catalytic performance evaluations in methane/ammonia reforming at “low” temperatures (≤650 °C in contrast to ≥ 1200 °C for the industry BMA process) demonstrated significant synergy between Ir and NbO_*x*_. The Ir-NbO_*x*_/SiO_2_ catalyst exhibited significantly higher activity (turnover frequency 8.5 s^–1^), selectivity (75%), and stability than the Ir/SiO_2_ analog, whereas the NbO_*x*_/SiO_2_ counterpart was almost inactive. Kinetic analysis suggested a Langmuir–Hinshelwood mechanism with an apparent activation energy of 65 kJ/mol. In contrast to ethane/ammonia reforming through C–C cleavage, the bimetallic Ir-NbO_*x*_/SiO_2_ was inefficient and less active than the Ir/SiO_2_, demonstrating tuned selectivity toward C–H activation rather than C–C cleavage. Notably, the SOMC-derived catalyst substantially outperforms the Ir-NbO_*x*_/SiO_2_-reference prepared by conventional calcination-reduction. This clearly demonstrates the advantage of the SOMC strategy: direct thermal decomposition of well-defined heterobimetallic precursors yields exceptionally high metal dispersion and strong electronic coupling between Ir and NbO_*x*_ species, which together underpin the enhanced catalytic performance. Moreover, the reaction-induced sintering of the Ir sites was inhibited in the presence of niobia, which can be explained by the strong interaction between the two species. By leveraging rational molecular-scale catalyst design, this work advances our understanding of heterobimetallic synergy in heterogeneous catalysis.

## Supplementary Material



## Notes

Experimental details (catalyst preparation, catalytic performance evaluation, and catalyst characterization); supporting catalytic and kinetic data; and characterization results (PDF)
