# Supplementary material for: Heterobimetallic Iridium-Niobia Catalyst for Efficient and Selective Methane Ammonia Reforming
Source: J Am Chem Soc. 2026 Jun 15;148(27):28507–17. doi: 10.1021/jacs.6c04917 (PMC13383725; doi:10.1021/jacs.6c04917)
Supplement: Supplementary file 1 [file ja6c04917_si_001.pdf]

Supporting Information

for

Heterobimetallic Iridium-Niobia Catalyst for Efficient and Selective Methane Ammonia Reforming

Jiachun Wu,[a,#] Zachary Dubrawski,[b,#] Shiwen Wu,[c] Samy Aïssiou,[b] Lingzhe Fang,[c] Laurent Veyre,[b] Chloé Thieuleux,[b] Tao Li,[d,e,]* Clément Camp[b,]* Yizhi Xiang[a,f,]*

a. Department of Chemical and Biomedical Engineering, University of Missouri, Columbia, Missouri 65211, United States. *yxpxb@missouri.edu

b. Laboratory of Catalysis, Polymerization, Processes and Materials (CP2M UMR 5128), CNRS, Universite Claude Bernard Lyon 1, CPE-Lyon, Institut de Chimie de Lyon, 43 Bd du 11 Novembre 1918, F-69616 Villeurbanne, France. *clement.camp@univ-lyon1.fr

c. Department of Chemistry and Biochemistry, Northern Illinois University, DeKalb, Illinois 60115, United States.

d. X-ray Science Division, Argonne National Laboratory, Lemont, Illinois 60439, United States

e. Department of Chemistry, Virginia Tech, Blacksburg, VA 24061, United States. *tli25@vt.edu

f. Materials Science and Engineering Institute, University of Missouri, Columbia, Missouri 65211, United States

Experimental

1. Catalysts Preparation

Unless otherwise noted, all reactions were performed either using standard Schlenk line techniques or in an MBRAUN glovebox under an atmosphere of purified argon (<1 ppm of O_2/H_2O). Glassware and cannulas were stored in an oven at ∼100 °C for at least 16 h prior to use. *n*-pentane was purified by passage through a column of activated alumina, dried over Na/benzophenone, vacuum-transferred to a storage flask, and freeze-pump-thaw degassed prior to use. The syntheses of Cp^*IrH_4[1] and $(Cp^*IrH_2)(Cp^*IrH_3)_2Nb(NMe_2)$[2] follow literature procedures. The SBA-15 silica support was synthesized[3] and dehydroxylated[4,5] at 700°C according to the literature procedures. Material **Ir/SiO$_2$** was prepared according to literature procedures[6] at a weight percent of 1% to match the value for the **Ir-NbO$_x$/SiO$_2$** material. Material **NbO$_x$/SiO$_2$** was prepared using the same experimental protocol described below for **Ir-NbO$_x$/SiO$_2$**, but using commercially available $Nb(NMe_2)_5$ as a reagent. H_2 gas was dried and deoxygenated over freshly regenerated R311G BASF catalyst/molecular sieves (4Å) prior to use. All other reagents were acquired from commercial sources and used as received.

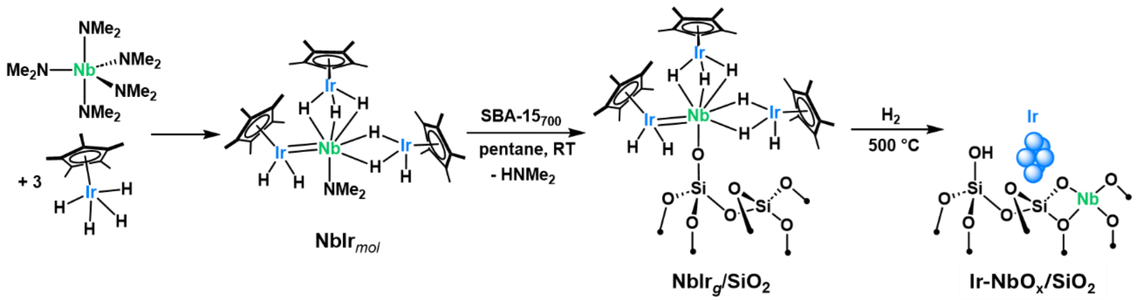

The Ir-NbO$_x$/SiO$_2$ was prepared according to the following steps. In an argon-filled glovebox, a 500 mL Schlenk flask was charged with 1.04 g freshly dehydroxylated SBA-15$_{700}$ along with a large Teflon coated stir bar. The silica was suspended in 200 mL of pentane, and, with rapid stirring, a solution of 21.0 mg of the complex (Cp*IrH$_2$)(Cp*IrH$_3$)$_2$Nb(NMe$_2$) (1.71 e-5 mol) in 5 mL of pentane was added dropwise over 5 minutes. The reaction was stirred at room temperature for 1 hour and allowed to decant, affording a colorless pentane supernatant and an orange solid. The solid was recovered by filtration and washed with copious volumes of pentane (3x 100 mL) before drying under vacuum (10^{-2} mbar) for several hours. The solid material was then transferred to a 454 mL glass reactor and further dried under high vacuum (10^{-6} mbar) for several hours. The argon atmosphere was then removed and replaced with 0.98 bar H_2. This reactor was then sealed and heated to 500°C overnight (18 h). The gaseous headspace was again evacuated, and the reactor was held under high vacuum for 1 hour, and then transferred into an argon glovebox for storage.

The Ir-NbO$_x$/SiO$_2$-ref sample was prepared through co-impregnation of the SBA-15 support using iridium(III) chloride and niobium(V) oxalate as the precursors. Specifically, 1 g of SBA-15 was impregnated with 20 mL of an aqueous solution containing appropriate amounts of the precursors to achieve a nominal Ir loading of 1 wt% and an Ir : Nb atomic ratio of 3. The mixture was stirred at room temperature for 2 h, after which the solvent was removed using a rotary evaporator at 60 °C under vacuum. The resulting powder was dried at 100 °C for 12 h and subsequently calcined under a dry air flow (200 mL/min) at 550 °C for 4 h to yield the final Ir-NbO$_x$/SiO$_2$-reference catalyst.

Pt/Al$_2$O$_3$ catalyst was prepared through impregnation of the Al$_2$O$_3$ support using Pt(NH$_3$)$_4$(NO$_3$)$_2$ as the precursor. Specifically, 1 g of Al$_2$O$_3$ was impregnated with 20 mL of an aqueous solution containing appropriate amounts of the precursors to achieve a nominal Pt loading of 1 wt%. The mixture was stirred at room temperature for 2 h, after which the solvent was removed using a rotary evaporator at 60 °C under vacuum. The resulting powder was dried at 100 °C for 12 h and subsequently calcined under a dry air flow (200 mL/min) at 550 °C for 4 h.

The Ir-NbO$_x$/SiO$_2$-TPO sample was prepared by treating Ir-NbO$_x$/SiO$_2$ sample under a flow of 20% O$_2$/Ar. The temperature was increased at a ramp of 10 °C/min to 550 °C and maintained at this temperature for 1 h.

The Ir-NbO$_x$/SiO$_2$-TPO/TPR sample was subsequently obtained by reducing the Ir-NbO$_x$/SiO$_2$-TPO sample under a flow of 10% H$_2$/Ar. The temperature was ramped at 10 °C/min to 650 °C and held for 30 min.

2. Catalysts Characterization

CO-DRIFT (Diffuse Reflectance Infrared Fourier Transform spectroscopy with CO as the probe molecule) was performed on a Thermo Scientific Nicolet Apex FTIR spectrometer equipped with a liquid-nitrogen-cooled mercury-cadmium-telluride (MCT) detector and a Praying Mantis high-temperature operando reaction chamber (Harrick Scientific Products Inc.) with CaF_2 windows. The powder samples of Ir-NbO_x/SiO_2 and Ir/SiO_2 were placed in the reaction chamber and pre-reduced at 650 °C under 10% H_2/Ar for 30 min. The sample was then cooled to 40 °C, purged with Ar until the background became stable. After that, the sample was exposed to 10% CO/Ar for chemisorption at 40 °C for 20 min. Finally, the spectra were collected after the sample was purged with Ar (for 10 min) to remove the gaseous CO.

Operando-DRIFT experiments were conducted using the same Apex FTIR spectrometer. Powder samples of Ir-NbO_x/SiO_2 and Ir/SiO_2 were loaded into the reaction cell and pre-reduced under a flow of 10% H_2/Ar at 650 °C for 30 min. After reduction, the temperature was lowered to 600 °C, and a background spectrum was collected under flowing Ar to represent a clean catalyst surface. The gas feed was then switched from Ar to a CH_4/NH_3/Ar mixture with partial pressure ratios of 1/1/2 at the same temperature and maintained for 30 min to ensure steady-state reaction conditions. Finally, the feed was switched back to Ar, and time-resolved spectra were recorded under Ar at 1-min intervals.

X-ray absorption spectroscopy (XAS) experiments were performed at beamline 12-BM of the Advanced Photon Source (APS) at the Argonne National Laboratory. The energy of the incident monochromatic X-rays was selected by using a water-cooled Si (111) double-crystal monochromator. The X-ray beam size is 500μm horizontal × 1000μ vertical. The energy resolution (ΔE/E) and photon flux (photos/sec) are 2×10^{-5} and 1×10^{11} @ 12 keV. The XANES spectroscopy was performed in fluorescence mode. The metal foil was used for energy calibration in each sample scan. The ex-situ samples were pressed into pallets and loaded into the sample holders. Three spectra were filtered before normalization (pre-edge normalization range, -150 to -60 eV; post-edge normalization range, 150 to 600 eV. The XAS data were collected at Nb K-edge or Ir L_3-edge in fluorescence mode. XAS data analysis was processed by Athena software.

X-ray photoelectron spectroscopy (XPS) experiments were performed on the Thermo Scientific Nexas G2 Surface Analysis System with a monochromated, micro-focused, low-power Al Ka X-ray source.

X-ray diffraction (XRD) experiments were performed on the Rigaku Ultima IV multipurpose X-ray diffraction (XRD) system.

Electron microscopy experiments were performed using the ThermoScientific Spectra 300 S/TEM at the Electron Microscopy Core (EMC) at the University of Missouri or the MET JEOL 2100F (FEG) microscope at the "Centre Technologique des Micro-structures", CTμ Villeurbanne, France.

Elemental analyses were performed under an inert atmosphere at Mikroanalytisches Labor Pascher, Germany. Elemental analysis for material Ir-NbO_x/SiO_2 (weight%) Ir 0.96, Nb 0.15, C 0.24, H 0.09, N 0.32.

3. Catalytic Performance Evaluation

The catalytic performance of methane-ammonia reforming was carried out in a home-built set-up. For each test, 10 mg of the catalyst was loaded into a quartz reactor with a volume of 2 mL (i.d., Φ = 1/2"). Before catalytic testing, the catalyst was activated in 10% H_2 balanced with Ar (100 mL/min) at 650 °C for 30 min. Then the spectra of Ar and the feed (by-passing the reactor), consisting of CH_4 (or C_2H_6) Ar, and NH_3 with desired partial pressure ratios, were collected for mass spectrometer calibration and used as a reference for calculating catalytic performance. The reactions were performed at 550-650 °C under atmospheric pressure, with space velocities of 480,000 or 960,000 mL/g_{cat}/h. Notably, the reaction was slightly limited by external mass transfer at a space velocity of 480,000 mL/g_{cat}/h (total flow 80 mL/min). Increasing the space velocity to 960,000 mL/g_{cat}/h eliminated external diffusion limitations; this condition was therefore used for the kinetic analysis shown in Figure 4 of the main text. The reactor effluent was analyzed using an online mass spectrometer (Leybold Inficon Transpector RGA TSP TH100). Specifically, m/z = 2, m/z = 15, m/z = 17, m/z =27, m/z = 28, m/z = 30, m/z = 40, and m/z = 41 were measured. The intensity of the m/z signal ($I_{m/z}$) is then converted to the intensity of each molecule ($I_{molecule}$) using a matrix based on the quantitative calibration. The MS intensities of different species were converted to partial pressures using an external standard calibration. The more detailed method for quantitative MS analysis is described in the reference.[7]

Specifically, the conversion and selectivity were calculated based on:

$$X = \frac{F_{i,in} - F_{i,out}}{F_{i,out}} \times 100\%$$

$$S_N = \frac{F_{HCN}}{F_{ammonia,\ in} - F_{ammonia,\ out}} \times 100\%$$

$$S_C = \frac{F_{i,out} \times n}{(F_{ethane,\ in} - F_{ethane,\ out}) \times 2} \times 100\%$$

where F_i is the mole flow rate and is calculated using the ideal gas equation of state ($P_i v = F_i RT$). The volumetric flow rate at the reactor exit was calibrated from changes in Ar partial pressure during the reaction. n represents the carbon number in the molecule of the product *i*.

The **chemical transient kinetic** experiment was performed at 650 °C over 20 mg of Ir-NbO_x/SiO_2 catalyst. In addition to the reactant mixture mentioned above, 3 mL/min Ne was used as an external standard to precisely calibrate the changes of volumetric flow rate due to the reaction/adsorption. The early-stage transient was induced by abruptly switching the influent gas from inert (Ar at 80 mL/min) to CH_4/NH_3/He (20/20/40 mL/min). The compositions of the reactor outlet during the transient experiments were continuously measured (every ~ 0.6 s) with the Inficon Transpector MPH Gas Analysis System.

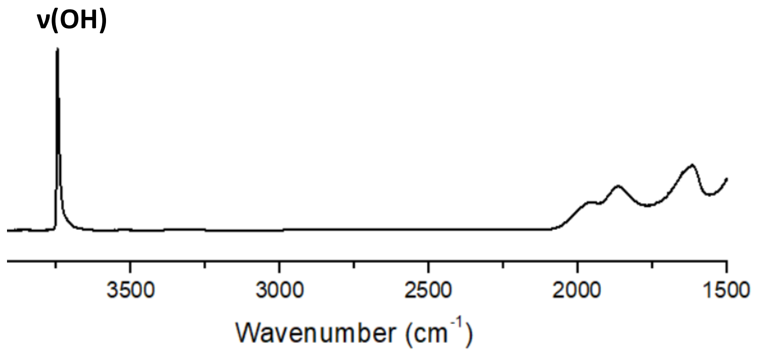

Figure S1. A: DRIFT spectrum of Ir-NbO_x/SiO_2, showing the isolated silanol stretching signal at 3748 cm^{-1} and the absence of C-H stretches (expected around 3000 cm^{-1}) after thermal reduction under hydrogen. Experimentally, the samples were prepared in a glovebox, sealed under argon in a DRIFT cell fitted with KBr windows, and then analyzed using a Nicolet 670 FT-IR spectrometer.

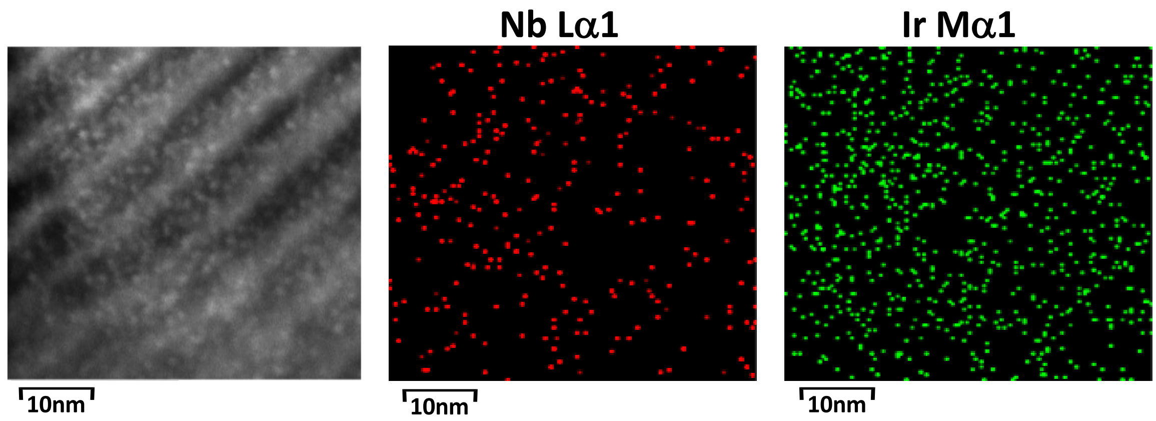

Figure S2. STEM-HAADF image of the Ir-NbO_x/SiO_2 catalyst with corresponding EDS elemental maps, showing a homogeneous distribution of both Nb and Ir components across the entire material, with no evidence of metal agglomeration or segregation.

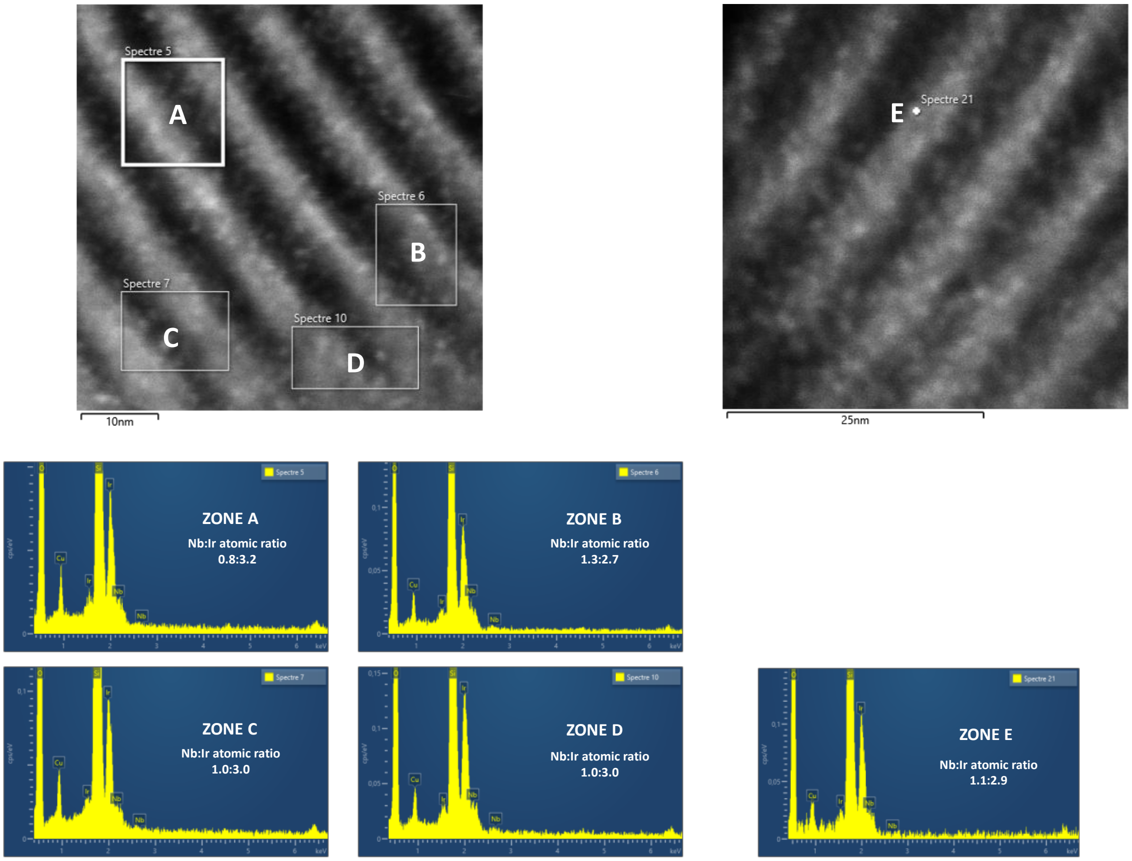

Figure S3. HAADF-STEM images of the Ir-NbO_x/SiO_2 material with corresponding EDS spectra acquired from multiple regions (A-E) of varying sizes, locations, and grain domains, along with the associated metal composition analysis. The EDS results consistently show a uniform Nb:Ir ratio across all zones, matching the expected 1:3 stoichiometry derived from the precursor nuclearity and corroborating the elemental analysis.

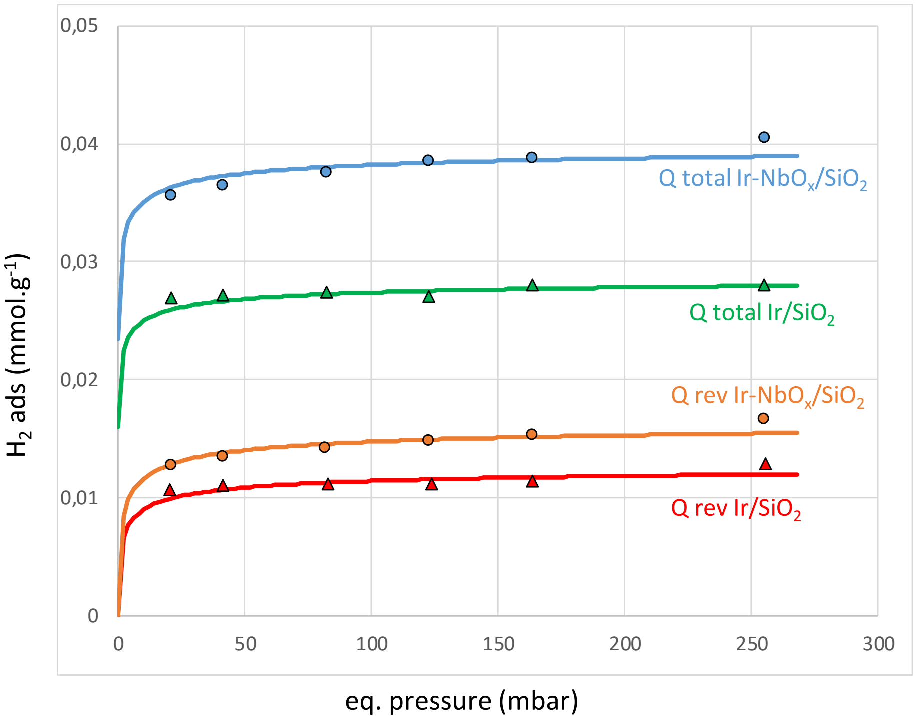

Figure S4. Total H_2 chemisorption isotherms at 298 K for Ir NPs for Ir-NbO_x/SiO_2 – experimental points (blue circles) and double Langmuir fit (blue curve) – and Ir/SiO_2 – experimental points (green triangles) and double Langmuir fit (green curve). Reversible H_2 chemisorption isotherms at 298 K for Ir NPs for Ir-NbO_x/SiO_2 – experimental points (orange circles) and double Langmuir fit (orange curve) – and Ir/SiO_2 – experimental points (red triangles) and double Langmuir fit (red curve). No measurable H_2 chemisorption is observed for NbO_x/SiO_2; thus, the chemisorption signal arises solely from the Ir component.

From the double Langmuir fit, we extracted the following parameter: H/Ir_{Total}, which translates the adsorbed atomic hydrogen *per* total iridium atoms. Then, we calculated the dispersion for Ir-NbO_x/SiO_2 and Ir/SiO_2 using the equation proposed by F. Drault *et al.*[8]: $D_{Ir}(\%)= a_Y\ (H/Ir)^5+ b_Y\ (H/Ir)^4+ c_Y\ (H/Ir)^3+ d_Y\ (H/Ir)^2+ e_Y\ (H/Ir)$ with a_Y=-2.116, b_Y=13.163, c_Y=-20.633, d_Y=-23.073, and e_Y=100.361 for iridium. Finally, we determined the Ir NPs size using the truncated cubic octahedron geometry. Using this strategy, we obtained the following values, which match perfectly the observation made by electron microscopy:

Material	Iridium loading (%wt)	Dispersion (M)	Avg. Nps size (nm)
Ir-NbO_x/SiO_2	1.0	80%	1.2
Ir/SiO_2	1.0	70%	1.4

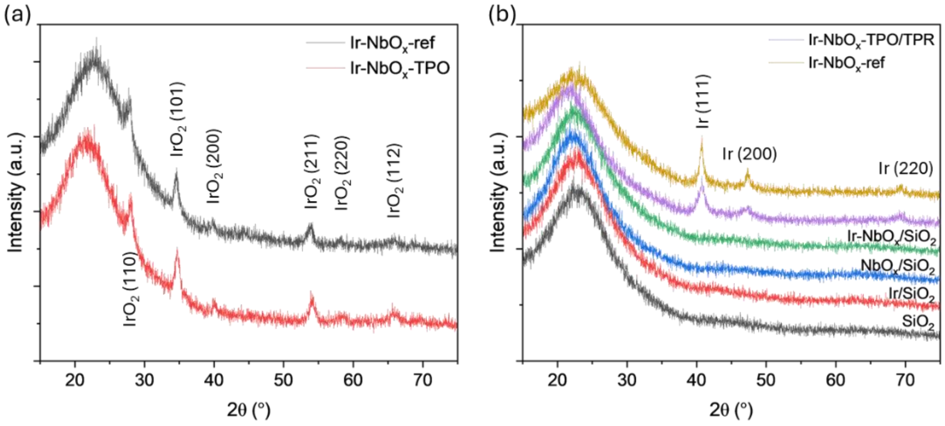

Figure S5. X-ray diffraction patterns. (a) Ir-NbO_x/SiO_2-reference (black line) before reduction and Ir-NbO_x/SiO_2-TPO (red line) after oxidation; (b) Ir-NbO_x/SiO_2 (green), Ir/SiO_2 (red), and NbO_x/SiO_2 (blue) in contrast to reduced Ir-NbO_x/SiO_2-reference after reduction (yellow) and Ir-NbO_x/SiO_2-TPO/TPR (purple).

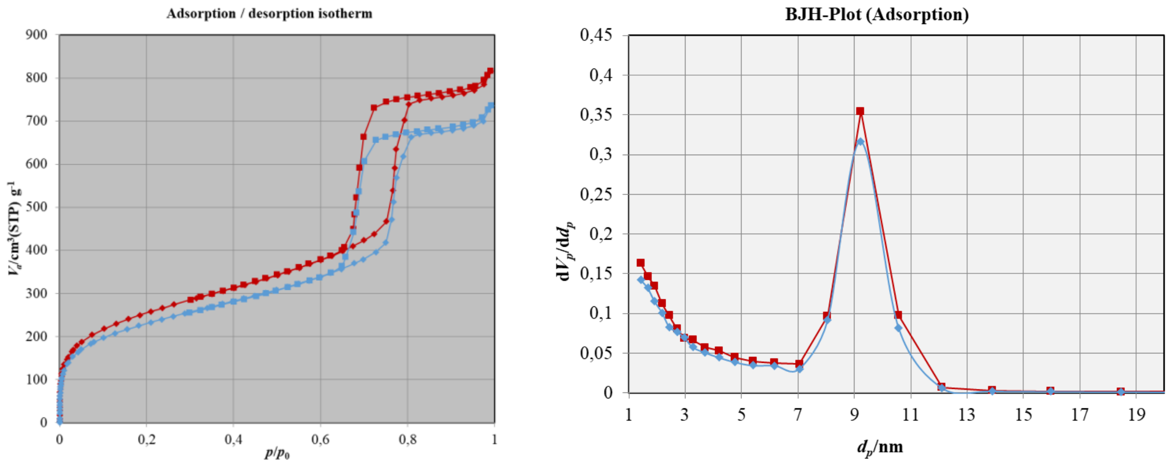

Figure S6. (Left) N_2 Adsorption/Desorption isotherm of the bare SBA-15$_{700}$ dehydroxylated support (red curve), as well as of catalyst Ir-NbO_x/SiO_2 (blue curve); (Right) BJH pore size distribution curve of the bare SBA-15$_{700}$ dehydroxylated support (red curve), as well as of catalyst Ir-NbO_x/SiO_2 (blue curve), derived from the adsorption branch of the nitrogen adsorption–desorption isotherm, showing in both cases a narrow distribution centered in the mesoporous range (identical in both cases, centered at 9.2 nm).

Summary of textural parameters, including specific surface area (BET method), total pore volume, and mean pore diameter (BJH method) calculated from the adsorption branch of the nitrogen adsorption-desorption isotherm, is shown below:

SBA-15$_{700}$

BET		
$a_{s,BET}$ (specific surface area)	931	[m^2 g^{-1}]
Total pore volume	1.3	[cm^3 g^{-1}]
BJH		
Plot data	Adsorption branch	
$d_{p,peak}$	9.2	[nm]

Ir-NbO_x/SiO_2

BET		
$a_{s,BET}$ (specific surface area)	839	[m^2 g^{-1}]
Total pore volume	1.1	[cm^3 g^{-1}]
BJH		
Plot data	Adsorption branch	
$d_{p,peak}$	9.2	[nm]

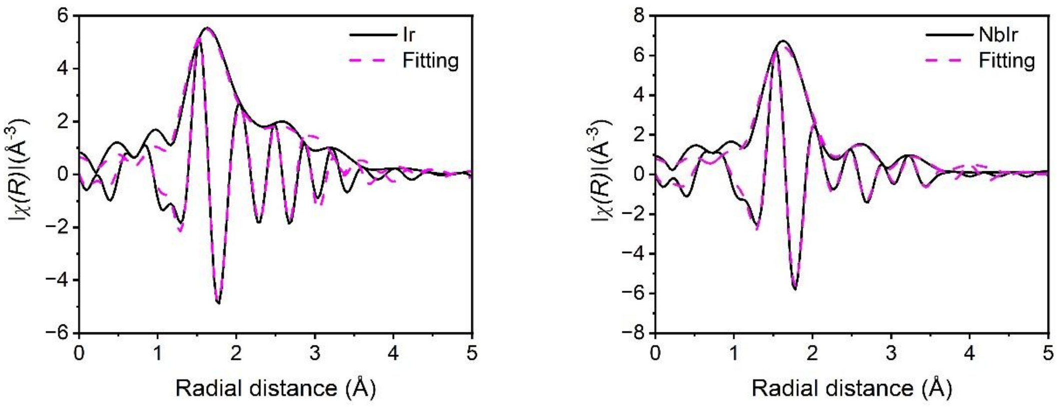

Figure S7. Ir L_{III}-edge EXAFS data, k^2-weighted, and fitting of used Ir/SiO_2 and $Ir\text{-}NbO_x/SiO_2$ catalysts.

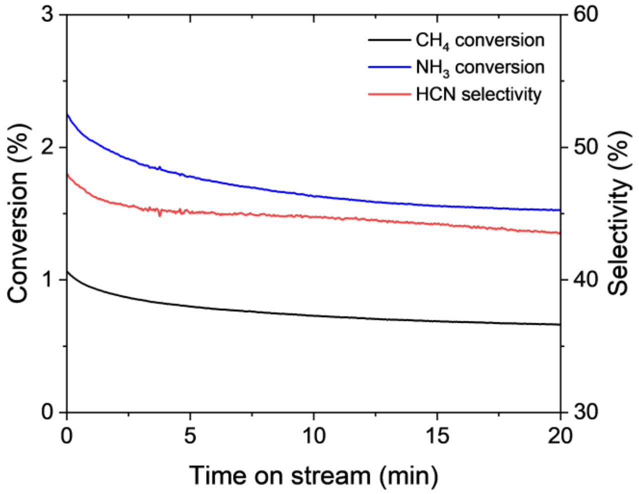

Figure S8. Catalytic performance of Pt/Al_2O_3 catalyst in methane/ammonia reforming. Reaction conditions: 650 ºC, $m_{catalyst}$ = 10 mg, space velocity 480,000 mL/g_{cat}/h, partial pressure of CH_4/NH_3/Ar: 0.25/0.25/0.5 atm.

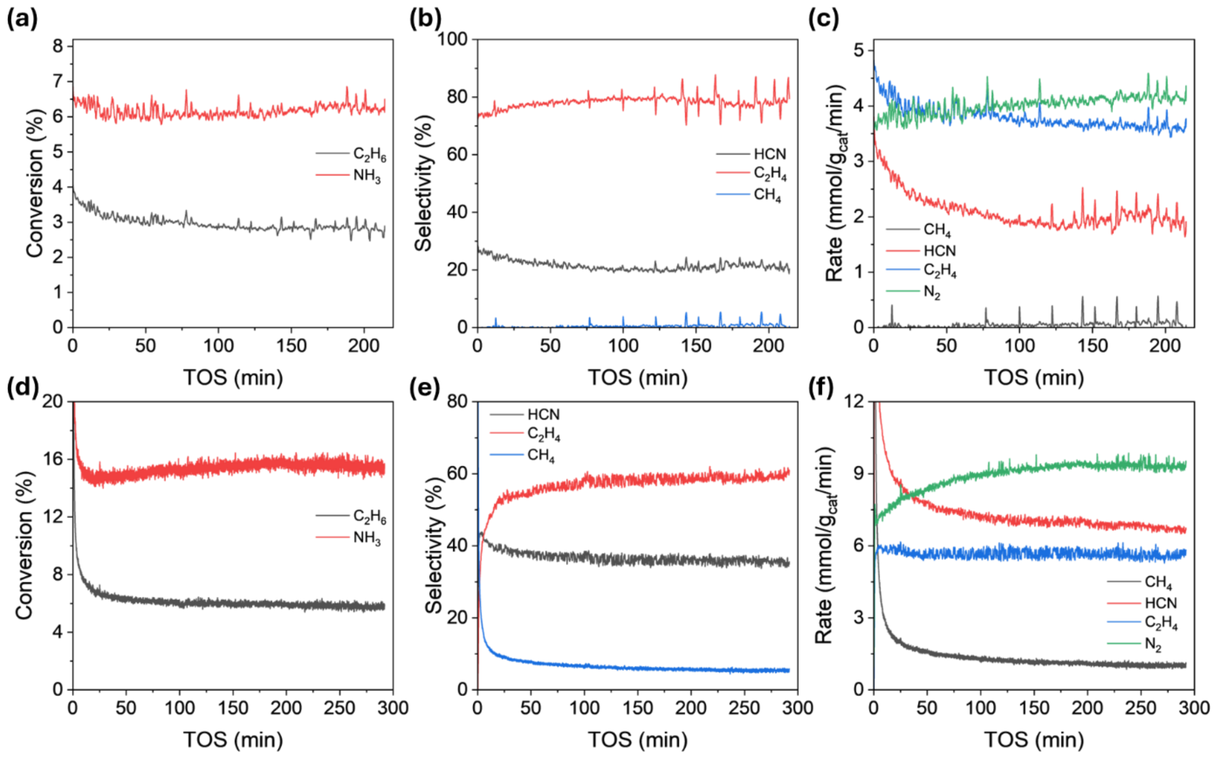

Figure S9. Ethane/ammonia reforming over the Ir-NbO_x/SiO_2 (a-c) and Ir/SiO_2 (d-f) catalysts. Reaction conditions: $m_{catalyst}$ = 10 mg, 650 ºC, space velocity 960,000 mL/g_{cat}/h, partial pressure of CH_4/NH_3/Ar: 0.25/0.25/0.5 atm.

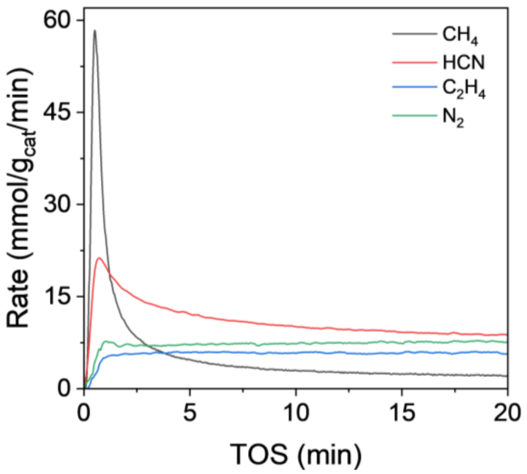

Figure S10. Early-stage rates of different products during ethane/ammonia reforming over the Ir/SiO_2 catalyst. Reaction conditions: $m_{catalyst}$ = 10 mg, 650 ºC, space velocity 960,000 mL/g_{cat}/h, partial pressure of CH_4/NH_3/Ar: 0.25/0.25/0.5 atm.

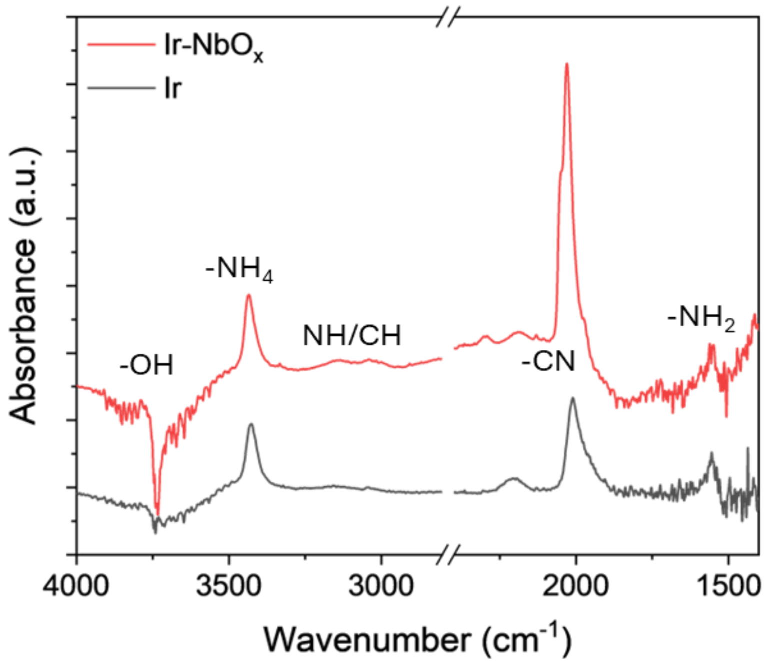

Figure S11. Operando-DRIFT spectra at 600 °C after switching from reactants (steady-state reaction conditions) to inert after 20 min. Top-red spectrum : Ir-NbO_x/SiO_2 ; bottom-black spectrum : Ir/SiO_2.

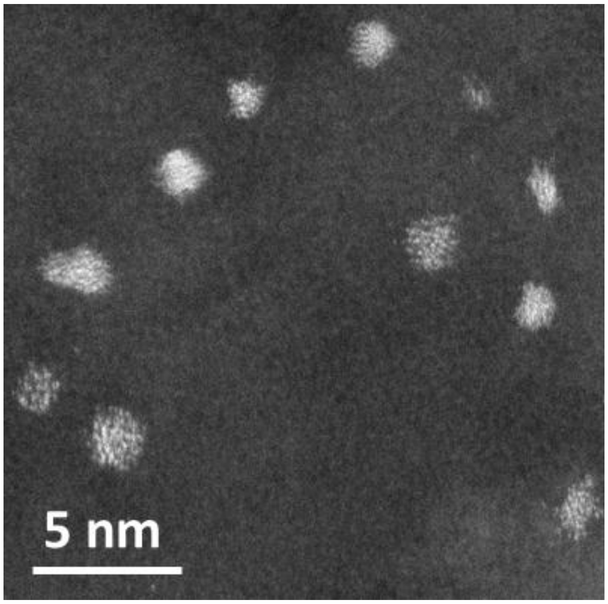

Figure S12. High-resolution HAADF-STEM image of the used Ir/SiO_2 catalyst.

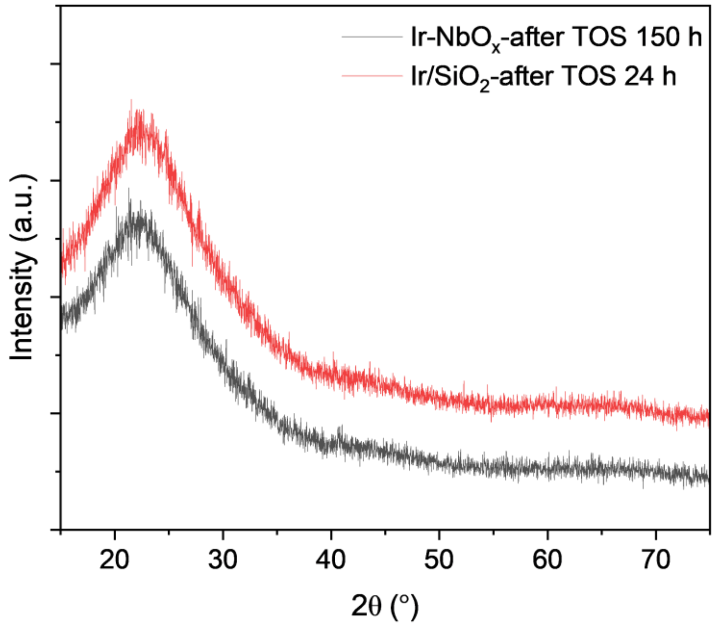

Figure S13. XRD patterns of the spent Ir/SiO_2 (red) and Ir-NbO_x/SiO_2 (black) catalysts.

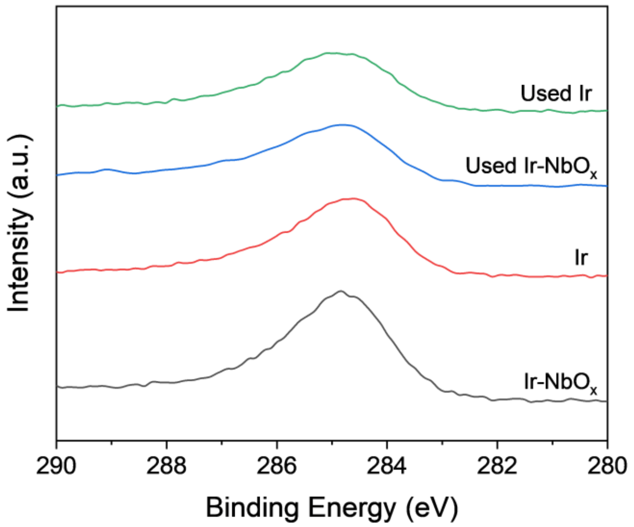

Figure S14. C 1s XPS spectra of the fresh and used Ir/SiO_2 and Ir-NbO_x/SiO_2 catalysts.

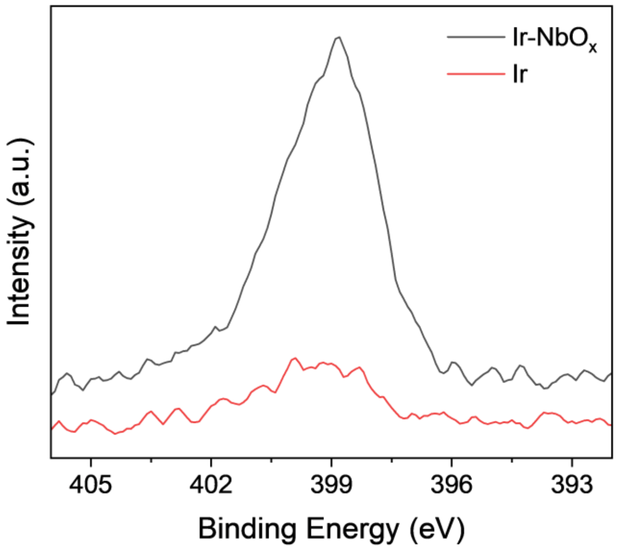

Figure S15. N 1s XPS spectra of the used Ir/SiO_2 and Ir-NbO_x/SiO_2 catalysts.

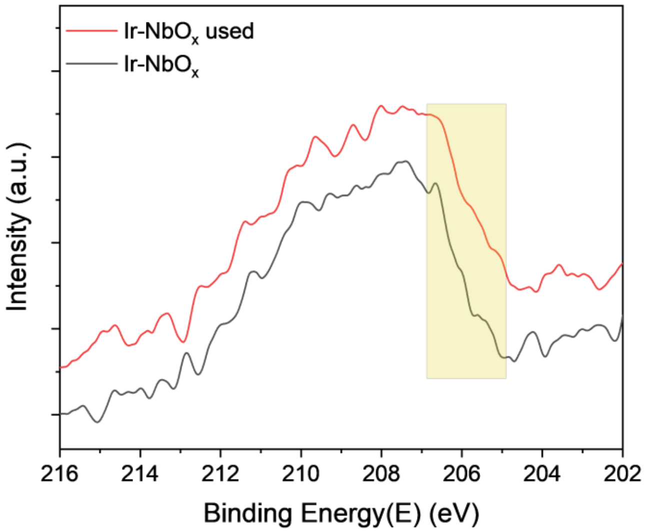

Figure S16. Nb 3d XPS spectra of fresh and spent Ir-NbO_x/SiO_2 catalyst.

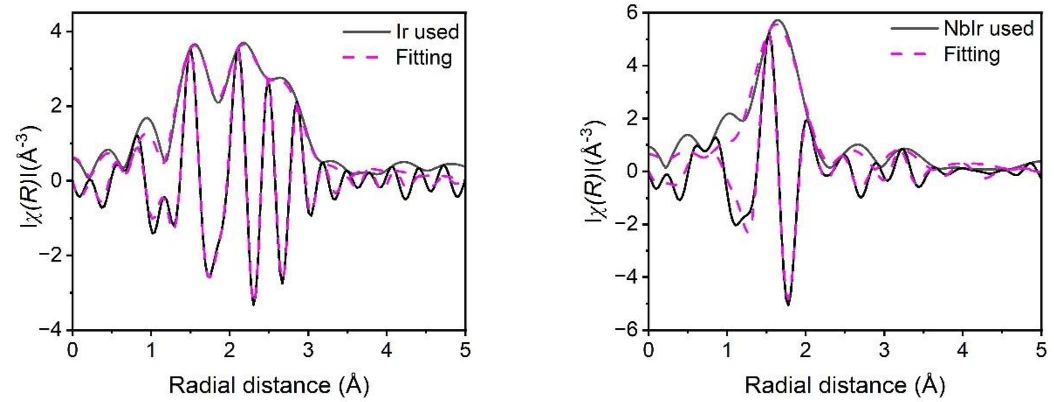

Figure S17. Ir L_{III}-edge EXAFS data, k^2-weighted, and fitting of used Ir/SiO_2 and Ir-NbO_x/SiO_2 catalysts.

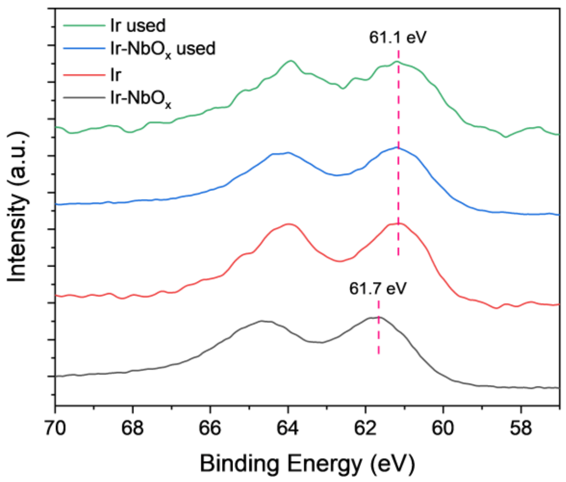

Figure S18. Ir 4f XPS spectra of fresh and spent Ir/SiO_2 and Ir-NbO_x/SiO_2 catalysts.

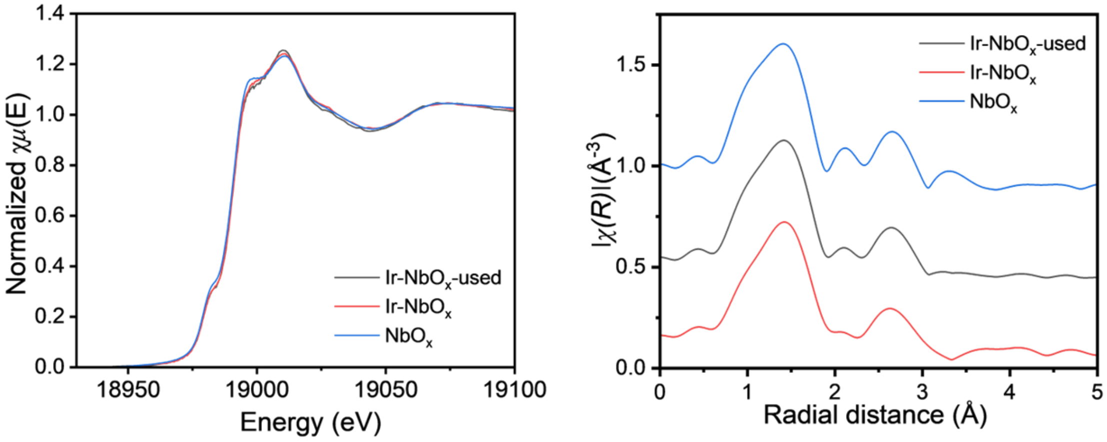

Figure S19. XANES and Fourier transform EXAFS spectra of fresh and spent Ir-NbO_x/SiO_2 and NbO_x/SiO_2.

Table S1. Fitting parameters of EXAFS spectra at Ir L_{III}-edge for Ir/SiO_2 and $NbIr/SiO_2$ catalysts.

Sample	Scattering path	CN	$\Delta\sigma^2 \times 10^3$ ($Å^2$)	ΔE_0 (eV)	R (Å)	R-factor
Ir/SiO_2 Reduced	Ir-O	4.3 ± 1.8	7.5 ± 6.5	13.1 ± 4.0	2.00 ± 0.03	0.011
	Ir-Ir	2.3 ± 3.9	3	13.1 ± 4.0	2.73 ± 0.14	0.011
Ir-NbO_x/SiO_2 Reduced	Ir-O	6.6 ± 0.2	10	12.1 ± 1.1	2.01 ± 0.01	0.005
	Ir-Ir	6.2 ± 0.8	15	12.1 ± 1.1	2.65 ± 0.01	0.005
Ir-NbO_x/SiO_2-ref	Ir-Ir	11.4± 0.4	3	7.8±1.0	2.71± 0.01	0.009
Ir-NbO_x/SiO_2-TPO	Ir-O	7.0± 0.4	3	10.2±1.5	1.98± 0.01	0.006
	Ir-Ir	4.3± 1.9	3	10.2±1.5	3.14± 0.03	0.006
Ir-NbO_x/SiO_2-TPO/TPR	Ir-Ir	10.5± 1.2	1.7 ± 0.9	6.5±1.1	2.7± 0.01	0.008
Ir/SiO_2 Used	Ir-O	2	6± 3	-1.6 ± 1.8	1.9 ± 0.02	0.004
	Ir-Ir	6.8 ± 0.4	10	-1.6 ± 1.8	2.61 ± 0.01	0.004
Ir-NbO_x/SiO_2 Used	Ir-O	5.3± 0.2	11	12.9 ± 1.4	2.01 ± 0.01	0.008
	Ir-Ir	4.8 ± 1	19	12.9 ± 1.4	2.66 ± 0.02	0.008

Table S2. Original data related to kinetic analysis in Figure 4(j) of the main text.

CH_4 flow (mL/min)	NH_3 flow (mL/min)	CH_4 conv (%)	NH_3 conv (%)	P_{CH4} (atm)	P_{NH3} (atm)	P_{HCN} (atm)	Experimental Rate (mmol/g/min)		Calculated Rate (mmol/g/min)		(Exp.-Cal.)^2	
							R_{HCN}	R_{N2}	R_{HCN}	R_{N2}	HCN	N_2
0	80		20	0.0000	0.4000	0.0000	0	32.7	0.0	27.6	0.00	26.46
2	80	25	14.6	0.0094	0.4270	0.0032	2.2	22.7	3.2	22.7	0.99	0.00
6	80	28.5	10	0.0268	0.4500	0.0099	6.9	15	8.0	18.1	1.17	9.33
10	80	31.5	11.8	0.0428	0.4410	0.0180	12.5	13	11.9	16.4	0.42	11.28
20	80	25.8	12.1	0.0928	0.4395	0.0298	20.7	10	20.1	11.3	0.37	1.81
40	80	17	12.5	0.2075	0.4375	0.0394	27.4	4.5	28.0	5.6	0.38	1.19
60	80	13	12	0.3263	0.4400	0.0432	30	4.9	29.7	3.1	0.11	3.30
40	10	5	35	0.2375	0.0406	0.0122	8.5	4	9.7	3.1	1.50	0.87
40	20	11	32	0.2225	0.0850	0.0236	16.4	5	17.2	5.5	0.71	0.27
40	30	15	28	0.2125	0.1350	0.0348	24.2	6	22.5	7.0	2.74	1.03
40	40	17	25	0.2075	0.1875	0.0373	25.9	7.5	25.7	7.5	0.05	0.00
40	50	17.7	20	0.2058	0.2500	0.0394	27.4	6.8	27.5	7.3	0.01	0.24
40	60	17.7	16.2	0.2058	0.3143	0.0398	27.7	6	28.2	6.8	0.27	0.57
40	80	17	11.5	0.2075	0.4425	0.0394	27.4	4.5	28.0	5.6	0.33	1.10

Table S3. Original data related to kinetic analysis in Figure 4(k) of the main text.

T (℃)	CH_4 conv (%)	NH_3 conv (%)	R_{HCN} (mmol/g/min)	R_{N2} (mmol/g/min)	P_{CH4} (atm)	P_{NH3} (atm)	k'_{HCN}	k'_{N2}
550	4.8	6.5	7.6	1.35	0.238	0.234	20941.4	10961.3
575	6.7	9	10.6	1.8	0.233	0.228	29357.3	14096.5
600	8.7	11.7	13.7	2.4	0.228	0.221	38166.2	18080.7
610	9.5	12.5	14.9	2.6	0.226	0.219	41574.6	19288.2
620	10.3	13.6	16.3	2.85	0.224	0.216	45596.6	20810.8
630	11	15	17.5	3.1	0.223	0.213	49134.2	22312.9
640	12.3	16.6	19.5	3.5	0.219	0.209	54956.1	24547.3
650	13	18	21.2	3.9	0.218	0.205	59982.6	26956.6

References

[1] T. M. Gilbert, F. J. Hollander, R. G. Bergman, *J. Am. Chem. Soc.* **1985**, *107*, 3508–3516.

[2] Z. Dubrawski, S. Aïssiou, E. Jeanneau, C. Thieuleux, C. Camp, *Dalt. Trans.* **2026**, *55,* 620–629.

[3] R. J. P. Corriu, Y. Guari, A. Mehdi, C. Reyé, C. Thieuleux, L. Datas, *Chem. Commun.* **2001**, *37*, 763–764.

[4] S. Lassalle, R. Jabbour, P. Schiltz, P. Berruyer, T. K. Todorova, L. Veyre, D. Gajan, A. Lesage, C. Thieuleux, C. Camp, *J. Am. Chem. Soc.* **2019**, *141*, 19321–19335.

[5] C. Coperet, A. Comas-Vives, M. P. Conley, D. P. Estes, A. Fedorov, V. Mougel, H. Nagae, F. Núñez-Zarur, P. A. Zhizhko, *Chem. Rev.* **2016**, *116*, 323–421.

[6] L. Escomel, D. Abbott, V. Mougel, L. Veyre, C. Thieuleux, C. Camp, *Chem. Commun.* **2022**, *58*, 8214–8217.

[7] S. Fadaeerayeni, X. Lyu, L. Fang, P. Wang, J. Wu, T. Li, T. Senftle, Y. Xiang, *J. Am. Chem. Soc.* **2024**, *146* (4), 2646–2653.

[8] F. Drault, C. Comminges, F. Can, L. Pirault-Roy, F. Epron, A. Le Valant, *Materials* (Basel), **2018**, 11(5), 819.
